# Phosphatase UBLCP1 is required for the growth, virulence and mitochondrial integrity of *Toxoplasma gondii*

**DOI:** 10.1186/s13071-025-06766-3

**Published:** 2025-03-28

**Authors:** Kaiyin Sheng, Kaiyue Song, Yimin Yang, Haiyan Wu, Zhendong Du, Xueqiu Chen, Yi Yang, Guangxu Ma, Aifang Du

**Affiliations:** 1 College of Animal Sciences, Institute of Preventive Veterinary Medicine, Zhejiang University, Hangzhou, 310058 China; 2 ZJU-Xinchang Joint Innovation Centre (TianMu Laboratory), Gaochuang Hi-Tech Park, Xinchang, 312500 China

**Keywords:** *Toxoplasma gondii*, UBLCP1, Mitochondrial integrity, Pathogenicity, Vaccine candidate

## Abstract

**Background:**

The mitochondrion is proposed as an ideal target organelle for the control of apicomplexan parasites, whose integrity depends on well-controlled protein import, folding, and turnover. The ubiquitin-like domain-containing C-terminal domain phosphatase 1 (UBLCP1) was found to be associated with the mitochondrial integrity in *Toxoplasma gondii*. However, little is known about the roles and mechanisms of UBLCP1 in this apicomplexan parasite.

**Methods:**

The subcellular localization of UBLCP1 in the tachyzoites of *T. gondii* was determined by an indirect immunofluorescence assay. The roles of UBLCP1 in the growth, cell cycle, and division of *T. gondii* were assessed by knocking out this molecule in the tachyzoites. Comparative phosphoproteomics between the UBLCP1-deficient and wild-type tachyzoites were performed to understand the roles of UBLCP1 in *T. gondii*. The virulence of UBLCP1-deficient tachyzoites of *T. gondii* was tested in mice.

**Results:**

UBLCP1 is expressed in the nucleus and cytoplasm of *T. gondii* tachyzoites. Tachyzoites lacking UBLCP1 exhibit collapsed mitochondrion, decreased mitochondrial membrane potential, and compromised growth and proliferation in vitro. Proteins involved in protein turnover and intracellular trafficking have been found differentially phosphorylated in the UBLCP1-deficient tachyzoites compared with the control. Deletion of UBLCP1 also shows that this phosphatase is essential for the propagation and virulence of *T. gondii* tachyzoites. Mice immunized with UBLCP1-deficient *T. gondii* tachyzoites survived challenges with the virulent PRU or VEG strain.

**Conclusions:**

UBLCP1 is required for the mitochondrial integrity and essential in the lytic cycle (e.g., host cell invasion and parasite replication) in vitro and the pathogenicity of this parasite in vivo. UBLCP1 is a candidate target for a vaccine or a drug for toxoplasmosis in animals.

**Graphical Abstract:**

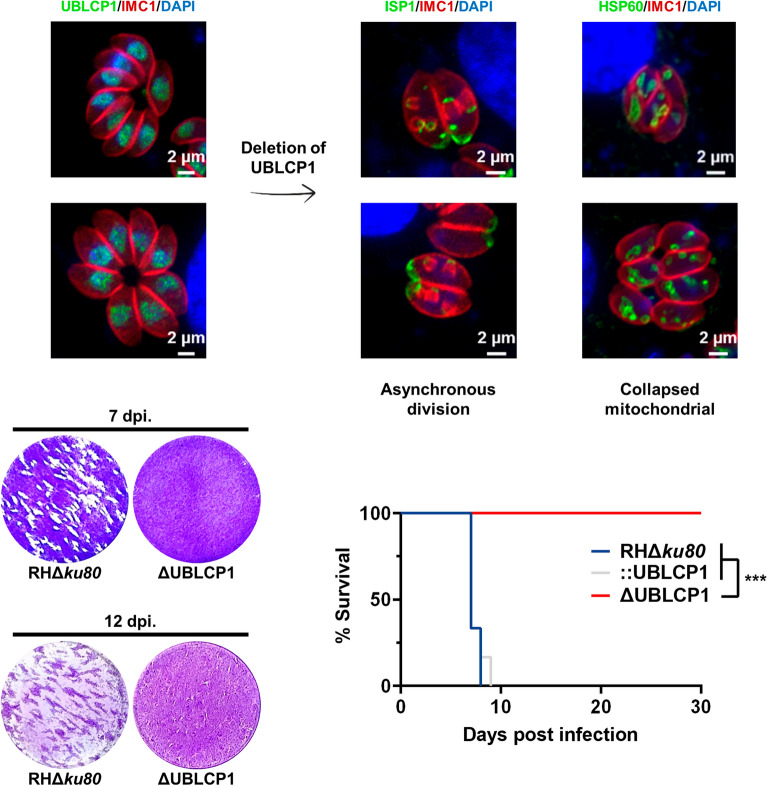

**Supplementary Information:**

The online version contains supplementary material available at 10.1186/s13071-025-06766-3.

## Background

The obligate intracellular apicomplexan parasite *Toxoplasma gondii* is arguably one of the most successful pathogens on the planet, infecting nearly all warm-blooded animals, including one-third of the human population [1, 2]. This unicellular parasite has a complex life cycle that goes through three infectious stages: the tachyzoite, the bradyzoite, and the sporozoite stages[3, 4]. *T. gondii* tachyzoites undergo rapid lytic cycles of host cell invasion, parasite replication, and egress from the infected cells, resulting in tissue destruction and consequent clinical symptoms (e.g., encephalitis, myocarditis, and even death) in immune-deficient individuals [5–7]. Owing to the insufficiency of the combination of pyrimethamine and sulfadiazine in toxoplasmosis treatment and a lack of vaccines, it is necessary to identify efficient drug targets or effective vaccine candidates to treat or prevent this disease in humans and animals.

The mitochondrion has been proposed as an ideal target organelle for the control of apicomplexan parasites. For instance, the antimalarial atovaquone targets the complex III (cytochrome bc1 complex) of *Plasmodium* species [8, 9]. The importance of the mitochondrion in the biochemical processes of *T. gondii* has been extensively reported, including redox, energy production, iron-sulfur cluster assembly, calcium and heme homeostasis, and other processes [10, 11]. Particularly, unlike mammalian cells, the intracellular *T. gondii* tachyzoite possesses only one mitochondrion which appears as a circular, “lasso-shaped” structure that loops around the nucleus [4, 12, 13], and the mitochondrial DNA (mtDNA) of this parasite encodes only fragmented ribosomal RNAs and three proteins (COX1, COX3, and CYTB) [14, 15]. However, *T. gondii* has abundant mitochondrial functions, such as iron-sulfur cluster assembly (ISC), coenzyme Q and heme biosynthesis, calcium homeostasis, and ATP production [16]. The morphological differences and functional conservation of mitochondrion between *T. gondii* and host animals stimulate biological investigations of the mitochondrial biology of *T. gondii* parasite.

The integrity and health of the mitochondrion depend on well-regulated protein import, folding, and turnover in cells [17–19]. Posttranslational modifications, such as phosphorylation and ubiquitination [20, 21], play crucial roles in regulating the import, folding, and turnover of mitochondrial proteins, determining the structure and function of mitochondrion in lifeforms, including *T. gondii* [22, 23]. In a genome-wide screening study, we observed mitochondrial abnormalities in the tachyzoites of *T. gondii* after deleting TGGT1_222010, a unique entry ID in the ToxoDB database. This gene is predicted to encode a homolog of ubiquitin-like domain-containing C-terminal domain phosphatase 1 (UBLCP1), which has been identified as the first specific 26S proteasome phosphatase [24–26]. However, little is known about the role of UBLCP1 in *T. gondii* (*Tg*UBLCP1), particularly in the structure and function of mitochondrion. Hence, in this study, we investigate the roles of UBLCP1 systematically in the growth, survival, and pathogenicity of tachyzoites (the acute infection stage) of *T. gondii*, to explore the essentiality of this phosphatase in the mitochondrial biology of this important parasite.

## Methods

### Parasites, host cells, animals, and antibodies

The type I RHΔ*ku*80Δ*hxgprt* strain (referred to as RHΔ*ku80*), type II PRU strain, and type III VEG strain of *T. gondii* used in this study were maintained as described previously [27]. Human foreskin fibroblast (HFF) cells were purchased from the Cell Bank of the Chinese Academy of Sciences (Beijing, China) and maintained as described elsewhere [28]. The 6-week-old Institute of Cancer Research (ICR) mice were obtained from the SLAC Laboratory Animal Co., Ltd. (Shanghai, China).

The primary rabbit anti-green fluorescent protein (GFP) antibody (Abclonal, China), polyclonal rabbit/mouse anti-IMC1 sera, polyclonal rabbit/mouse anti-GAP45 sera, polyclonal rabbit/mouse anti-ISP1 sera, polyclonal mouse anti-TOM40 sera, polyclonal rabbit anti-HSP60 sera, polyclonal rabbit anti-F1β ATPase sera (prepared and preserved in the lab; [29–33]), and secondary horseradish peroxidase (HRP)-conjugated goat anti-rabbit (or mouse) immunoglobulin (Ig)G (H + L) (Fude, China), and Alexa Fluor 488 (or 594) goat anti-mouse/rabbit IgG (Invitrogen, USA) were purchased and used according to the manufacturer’s instructions, or prepared internally as described previously [34].

### Construction of stable transgenic parasites

Transgenic parasites were constructed from *T. gondii* RHΔ*ku80* tachyzoites, as described previously [35, 36]. For hemagglutinin (HA) epitope-tagged strains of genes of UBLCP1, the parental RHΔ*ku80* tachyzoites were transfected with a hypoxanthine xanthine guanosine phosphoribosyl transferase (HXGPRT; amplified from the pLinker-BirA-6HA-HXGPRT-loxP plasmid) resistance cassette and specific CRISPR plasmid via electroporation. Guide RNAs (gRNAs) were designed using EuPaGDT (http://grna.ctegd.uga.edu/) [37] and cloned into the pSAG1::Cas9-U6::sg*Bbs* I plasmids via the *Bbs* I restriction site. Cassette integration of target gene was selected for 7 days in a medium supplemented with mycophenolic acid and xanthine, which was subsequently verified by polymerase chain reaction (PCR). For the UBLCP1 overexpressed strain, the coding sequence of TGGT1_222010 was PCR amplified from RHΔ*ku80* cDNA using KOD One PCR Master Mix (Toyobo, Tokyo, Japan), following the manufacturer’s instructions. The PCR product was ligated into the pTubulin-EGFP plasmid as described previously [27]. The donor fragment amplified from the pTubulin-UBLCP1-EGFP was imported into the parental RHΔ*ku80* together with plasmid pSAG1::CAS9-U6::sgUPRT (Addgene plasmid 54467). Recombinant *T. gondii* populations were selected with 10 μM 5-fluoro-2-deoxyuridine (FUDR) (Sigma-Aldrich, USA) and confirmed by PCR and immunofluorescence assay (IFA) amplification. The UBLCP1 gene complementary strain (::UBLCP1) was constructed using the same strategy, although the donor fragment was amplified from the pTubulin-UBLCP1-EGFP, and the plasmids pSAG1::CAS9-U6::sgUPRT were transfected into the ΔUBLCP1 strain. For deficient strains, the open reading frame of UBLCP1 was replaced by a dihydrofolate reductase (DHFR) resistance cassette. Stable integration was selected in 3 μM pyrimethamine (Sigma-Aldrich, USA) for at least 7 days, whereas the screening time was extended to 14 days due to the significant growth inhibition after UBLCP1 deletion. After that the positive single clone was isolated by limiting dilution and verified using PCR and quantitative PCR (qPCR) amplification analysis. Targeted deletion of the coding region was validated in PCR 1, correct targeted 5′ integration was validated in PCR 2, and correct targeted 3′ integration was validated in PCR 3. All the primers used in this study are listed in Supplementary Table S2.

### Western blot

Completely egressed tachyzoites of *T. gondii* were collected and lysed in ice-cold radioimmunoprecipitation assay (RIPA) lysis buffer (Beyotime Biotechnology, China) supplemented with protease and phosphatase inhibitor cocktails (Bimake, USA), and they were incubated at 4 °C for 30 min to generate protein lysate. After 100 ℃ denaturation, protein lysate was subjected to polyacrylamide gel electrophoresis and then transferred onto a polyvinylidene fluoride (PVDF) membrane (Millipore, USA). The PVDF membrane was blocked with 5% (*w*/*v*) skimmed milk and then incubated with primary and secondary antibodies. Signals were visualized by a standard chemiluminescent HRP method with the ECL reagent (Fude, China), and detected using the ChemiDoc chemiluminescence system (Bio-Rad, Hercules, USA).

### IFA

Monolayer HFFs on a 24-well coverslip were infected with 10^5^ *T. gondii* tachyzoites and incubated for 24 h, then fixed with 4% (*w*/*v*) paraformaldehyde (PFA) for 15 min. Following permeabilization with 0.25% (*v*/*v*) Triton X-100, the sample was blocked with 1% (*w*/*v*) bovine serum albumin (BSA) for 30 min. After incubation with primary and secondary antibodies, cell nuclei were stained with 4,6-diamidino-2-phenylindole (DAPI; Sigma, USA). Finally, the coverslips were inverted in 50% glycerol on a glass slide, and images were acquired under a Zeiss LSM880 confocal laser scanning microscope equipped with an Airyscan system (Zeiss, Germany). Fluorescent signals were used to indicate the subcellular localization of proteins, cell division of tachyzoites, and/or mitochondrial morphology as indicated [38].

### Plaque assay

The plaque assay was performed as previously described [39]. In brief, confluent HFFs in six-well cell plates were infected with 200 freshly purified tachyzoites of RHΔ*ku80* or ΔUBLCP1 and cultured for 7 or 12 days. After washing three times in PBS to remove broken cells and overflowing tachyzoites, the remaining cells were fixed with methanol and stained with 0.2% crystal violet for 30 min. All plaque assays were performed in triplicate.

### Intracellular replication assay

Intracellular replication assay was carried out as described elsewhere [28]. Owing to a growth inhibitory effect, about 1 × 10^5^ tachyzoites of the UBLCP1-deficient strain were collected and then passed into HFFs with the parental RHΔ*ku80* under normal conditions (37 °C with 5% CO_2_) for subsequent experiments. Monolayer HFFs on 24-well coverslips were infected with 1 × 10^5^ freshly egressed tachyzoites, washed with fresh nonresistant 2% FBS medium 3 h postinfection to remove extracellular parasites, and incubated for 21 h. After precooled methanol fixation and 1% BSA blocking, cells were incubated with rabbit anti-GAP45 sera to detect individual parasites of parasitophorous vacuoles (PVs), followed by incubation with Alexa Fluor 594 goat anti-rabbit IgG. Nuclei were stained with DAPI. The parasites were cultured for a longer period compared with the normal replication assay, and then the asynchronous division rate of indicated strains in PVs was recorded at 32 h postinfection as previously described [38]. At least ten images for each sample were acquired stochastically with a Zeiss LSM880 confocal laser scanning microscope (Zeiss, Germany) to count the numbers of parasites and host nuclei.

### Invasion assay

For invasion assay, 5 × 10^6^ freshly harvested tachyzoites of the parental RHΔ*ku80* or ΔUBLCP1 strain were inoculated on monolayer HFFs on the 24-well coverslips and allowed to invade for 30 min. After 4% PFA fixation and 1% BSA blocking, cells were incubated with mouse anti-*Tg* sera and Alexa Fluor 594 goat anti-mouse IgG. After washing three times in PBS, the cells were then permeabilized with 0.25% Triton X-100 and blocked with 1% BSA, followed by the labeling of all parasites with rabbit anti-GAP45 and incubation with Alexa Fluor 488 goat anti-rabbit IgG. Nuclei were stained with DAPI. At least ten images for each sample were taken to count the number of parasites and host nuclei for the attachment and invasion assay. The parasite invasion efficiency was calculated as described previously [40].

### Cell cycle analysis

Cell cycle assay was carried out as previously described [41]. In brief, tachyzoites of the parental RHΔ*ku80* or ΔUBLCP1 strain were cultured in HFF cells for 48 h under normal growth conditions (37 °C with 5% CO_2_). Then, the parasites were released from cells and purified with filters, followed by washing in PBS. The harvested tachyzoites were fixed with precold 70% ethanol at −20 ℃ overnight and subsequently stained with propidium iodide (Beyotime Biotechnology, China). The signal intensity of propidium iodide staining was collected by the CytoFLEX flow cytometer (BD Biosciences, USA). At least 10,000 tachyzoites were recorded and analyzed for each strain in each experiment.

### TUNEL assay

Terminal deoxyribonucleotide transferase (TdT)-mediated dUTP nick end labeling (TUNEL) assay was performed as described elsewhere [41]. Briefly, 24-well coverslips were coated with 0.01% poly-l-lysine, followed by inoculation with purified tachyzoites of the parental RHΔ*ku80* or ΔUBLCP1 strains. Parasites were fixed with 4% PFA, permeabilized with 0.25% Triton X-100, and then incubated in the TUNEL reaction mix (Beyotime Biotechnology, China). Rabbit anti-IMC1 sera and Alexa Fluor 488 goat anti-rabbit IgG were used as primary and secondary antibodies to indicate the inner membrane complex of *T. gondii* tachyzoites under a Zeiss LSM880 confocal laser scanning microscope. Purified tachyzoites were also incubated in the TUNEL reaction mix and identified by the CytoFLEX flow cytometer to evaluate the DNA damage rate of indicated strains.

### TEM

Freshly harvested *T. gondii* tachyzoites were fixed with 2.5% glutaraldehyde at 4 °C overnight and embedded with agar. The parasites were fixed with 1% osmium acid for 2 h, followed by gradient dehydration treatment of ethanol (30%, 50%, 70%, and 80% ethanol) and acetone solution (90% and 95% acetone solution). The dehydration-treated samples were permeated with a mixture of Spurr embedding agent and acetone, and then subsequently into a pure Spurr embedding agent overnight. The permeation-treated samples were embedded, heated at 70 °C overnight, and cut into ultrathin sections on a Leica EM UC7 (Leica Microsystems, Germany). After staining with lead citrate and uranium dioxide acetate, the ultrathin sections of the parental RHΔ*ku80* and ΔUBLCP1 strains tachyzoites were observed under a Hitachi H07560 transmission electron microscope (Hitachi, Japan) to demonstrate the ultrastructure of the mitochondrion.

### Mitochondrial morphology measurement

To observe the mitochondrial morphology of indicated strains, IFA assay was performed as mentioned above. HFFs infected with *T. gondii* tachyzoites were incubated with polyclonal mouse anti-TOM40 sera (1:1,000; indicating mitochondrial outer membrane), polyclonal rabbit anti-HSP60 sera (1:1,000; indicating mitochondrial matrix), or polyclonal rabbit anti-F1β ATPase sera (1:1,000; indicating mitochondrial inner membrane) as primary antibodies, along with Alexa Fluor 488 goat anti-rabbit/mouse IgG as secondary antibodies. Additionally, rabbit/mouse anti-IMC1 sera and Alexa Fluor 594 goat anti-rabbit/mouse IgG incubated with the samples were used for *T. gondii* tachyzoites identification. To count the mitochondrial abnormal rate, the RHΔ*ku80* and ΔUBLCP1 tachyzoites in PVs were recorded at 32 h postinfection, after incubating with polyclonal rabbit anti-HSP60 sera along with Alexa Fluor 488 goat anti-rabbit IgG. At least ten images for each sample were acquired stochastically with a Zeiss LSM880 confocal laser scanning microscope (Zeiss, Germany).

### Mitochondrial membrane potential measurement

Tachyzoites of parental RHΔ*ku80* or ΔUBLCP1 strains were cultured in cells for 2 or 3 days under normal growth conditions. Then 1 × 10^7^ freshly egressed tachyzoites at least were harvested from cells and incubated in JC-1 staining buffer (Solarbio, China) at 37 °C for 20 min. Referring to a previous study [42], the fluorescence signals were collected by the CytoFLEX flow cytometer (BD Biosciences, USA) and quantified by the FlowJo software to compare the mitochondrial membrane potential of the parental RHΔ*ku80* and ΔUBLCP1 tachyzoites.

### Phosphatase activity assay

Phosphatase activity of UBLCP1 was detected using a serine/threonine phosphatase assay system (Promega, USA) as previously described [43, 44]. Prokaryotic expression plasmids of UBLCP1 and UBLCP1-D470A were constructed and transformed into *Escherichia coli* Rosetta. Then, the cells were induced with 1 mM isopropyl-β-d-thiogalactopyranoside (IPTG) at 16 °C for 24 h to express. Cells were collected by centrifugation at 8000*g* for 5 min and then solubilized in lysis buffer (25 mM tris–HCl, pH 7.5, 300 mM NaCl, 1 mM phenylmethanesulfonyl fluoride, EDTA-free protease inhibitor cocktail, and 1 mg/ml lysozyme), followed by sonicated extraction of protein. The supernatant obtained by centrifugation at 12,000*g* for 20 min (4 °C) was incubated with Ni–NTA agarose resin for 2 h. After washing twice with washing buffer (25 mM tris–HCl, 300 mM NaCl, and 20 mM imidazole), proteins were eluted with elution buffer (25 mM tris–HCl, 300 mM NaCl, and 250 mM imidazole). Recombinant proteins were analyzed by sodium dodecyl-sulfate polyacrylamide gel electrophoresis (SDS-PAGE) and immunoblotted using a His-tag antibody as probe.

For the in vitro phosphatase assay, protein concentrations were measured with the Enhanced BCA Protein Assay Kit (Beyotime, P0010, China). Phosphatase activity was measured in phosphatase buffer (50 μL reaction mixtures containing 50 mM imidazole, pH 7.2, 5 mM MgCl_2_, 0.2 mM EGTA, 0.02% BME, and 0.1 mg/mL BSA). The protein serine/threonine phosphatase activity of recombinant protein UBLCP1 and mutant protein UBLCP1-D470A was measured against phosphothreonyl peptides, RRApTVA, using a protein phosphatase assay system, according to the manufacturer’s protocol.

### Phosphoproteomics

Tachyzoites of the parental RHΔ*ku80* and ΔUBLCP1 strains were passed into HFFs under normal conditions (37 °C with 5% CO_2_) for 2–3 days until the parasites were about to egress. Intracellular parasites were released, purified, and lysed in 8 M urea supplemented with the protease inhibitor cocktail (Calbiochem, Germany) and phosphatase inhibitor cocktail (Millipore, USA). After sonication for 3 min (sonication for 3 s, pause for 5 s, 25% power, power 220 W) and centrifugation at 12,000*g* for 10 min at 4 °C, the protein concentration of the supernatant was assayed using a BCA method. Dithiothreitol (DTT, final concentration: 5 mM; Sigma-Aldrich, USA) was added into the protein samples for reduction at 37 °C for 60 min, followed by incubation with iodoacetamide (final concentration: 15 mM; Sigma-Aldrich, USA) at room temperature for 15 min in dark. After that, the urea concentration of the samples was diluted to less than 1 M with 50 mM TEAB (triethylammonium bicarbonate); then, the protein samples were digested with Trypsin at 1:50 (*w*/*w*) (Promega, USA) overnight. The digested samples were desalted using a Bond Elut C18 SPE column (Agilent, USA) and then dried for use. Peptide mixtures were first incubated with titanium dioxide (TiO_2_) with vibration in an enrichment buffer (50% acetonitrile, 1 M lactic acid, 5% trifluoroacetic acid). The suspensions were sequentially washed with 50% acetonitrile and 5% trifluoroacetic acid and 50% acetonitrile and 0.1% trifluoroacetic acid. The enriched phosphopeptides were then eluted with vibration in a buffer containing 10% NHOH, followed by collection and vacuum lyophilization. The enriched phosphopeptides redissolved in ultrapure water were centrifuged using C18 columns and eluted in an aqueous solution containing 4–31% acetonitrile (pH 9.0) at concentration gradient intervals of 1.5%. The classified phosphopeptides were combined into six components and freeze-dried in a vacuum for liquid chromatography–mass spectrometry (LC–MS) analysis.

The enriched peptides were dissolved in 0.1% (*v*/*v*) formic acid (Fluka, Germany) and sequentially separated on an EASY-nLC 1000 system (Thermo Fisher Scientific, USA) using solvent A (0.1% formic acid and 2% acetonitrile) and solvent B (0.1% formic acid and 90% acetonitrile). The electrospray voltage applied was 2.0 kV. The *m*/*z* scan range was 400–1800 m/z for the full scan, and the scanning resolution was set to 70,000. Automatic gain control (AGC) was set at 1E5. The signal threshold was set to 20,000 ions/s.

For bioinformatics analysis, the raw data were processed and searched using the MaxQuant search engine (v.1.6.15.0) [45]. With reference to the *T. gondii* genome in the ToxoDB database (https://toxodb.org), differentially phosphorylated proteins were identified with the threshold set as fold-change (FC) > 1.5 or < −1.5 and *P* < 0.05. Subcellular localization of differentially phosphorylated proteins was predicted according to the spatial data obtained from WolF Psor. The Cluster of Orthologous Genomics (COGs) functional classification [46] of the differentially phosphorylated proteins was identified by comparison with the parasite genome in the ToxoDB database (https://toxodb.org).

### Animal experiments

The 6-week-old ICR mice (*n* = 6) were injected intraperitoneally (i.p.) with 100 tachyzoites of RHΔ*ku80* (parental strain), ΔUBLCP1 (deletion mutant strain), or ::UBLCP1 (complementary strain). The infected mice were raised under well-controlled conditions and monitored daily for signs suggestive of *T. gondii* infection for 2 weeks. Each mouse was also infected i.p. with 1,000, 10,000, or even 100,000 ΔUBLCP1 tachyzoites to assess the degree of virulence attenuation of ΔUBLCP1. Mice that survived at 30 days postinfection were euthanized using 2% isoflurane.

Parasite burden in peritoneal fluids was measured as described previously [47]. In brief, 6-week-old ICR mice (*n* = 3) were infected i.p. with 100 tachyzoites of RHΔ*ku80* or ΔUBLCP1. A total of 5 days postinfection, the mice were euthanized as described above; their peritoneal fluids were collected and used to extract genomic DNA for quantitative PCR amplification of the β-tubulin gene of *T. gondii*. Quantification of *T. gondii* tachyzoites in the collected peritoneal fluid was performed on the basis of a standard curve of threshold cycle (Ct) values of the β-tubulin gene for 2 mL peritoneal fluid of blank mice supplemented with 0, 10^0^, 10^1^, 10^2^, 10^3^, 10^4^, 10^5^, 10^6^, or 10^7^ RHΔ*ku80* tachyzoites [47].

To evaluate immune protection against acute infection in mice elicited by ΔUBLCP1 vaccination, 6-week-old ICR mice were (*n* = 6) preinfected i.p. with 10^4^ tachyzoites of ΔUBLCP1 strain and challenged with 10^4^ tachyzoites of the RHΔ*ku80*, VEG, or PRU strain 30 days post the initial infection. PBS was used as the control. Feeding, clinical symptoms, and survival observations were monitored for 30 days post-reinfection.

### Statistical analysis

Statistical analysis was performed with GraphPad Prism (GraphPad, USA). One-way analysis of variance (ANOVA), two-way analysis of variance (ANOVA), or unpaired two-tailed Student’s *t*-test was used to assess the statistical differences between or among groups. The statistically significant level was set as *P* ≤ 0.05.

## Results

### Phosphatase UBLCP1 is expressed in the nucleus and cytoplasm in *T. gondii*

**Fig. 1:**
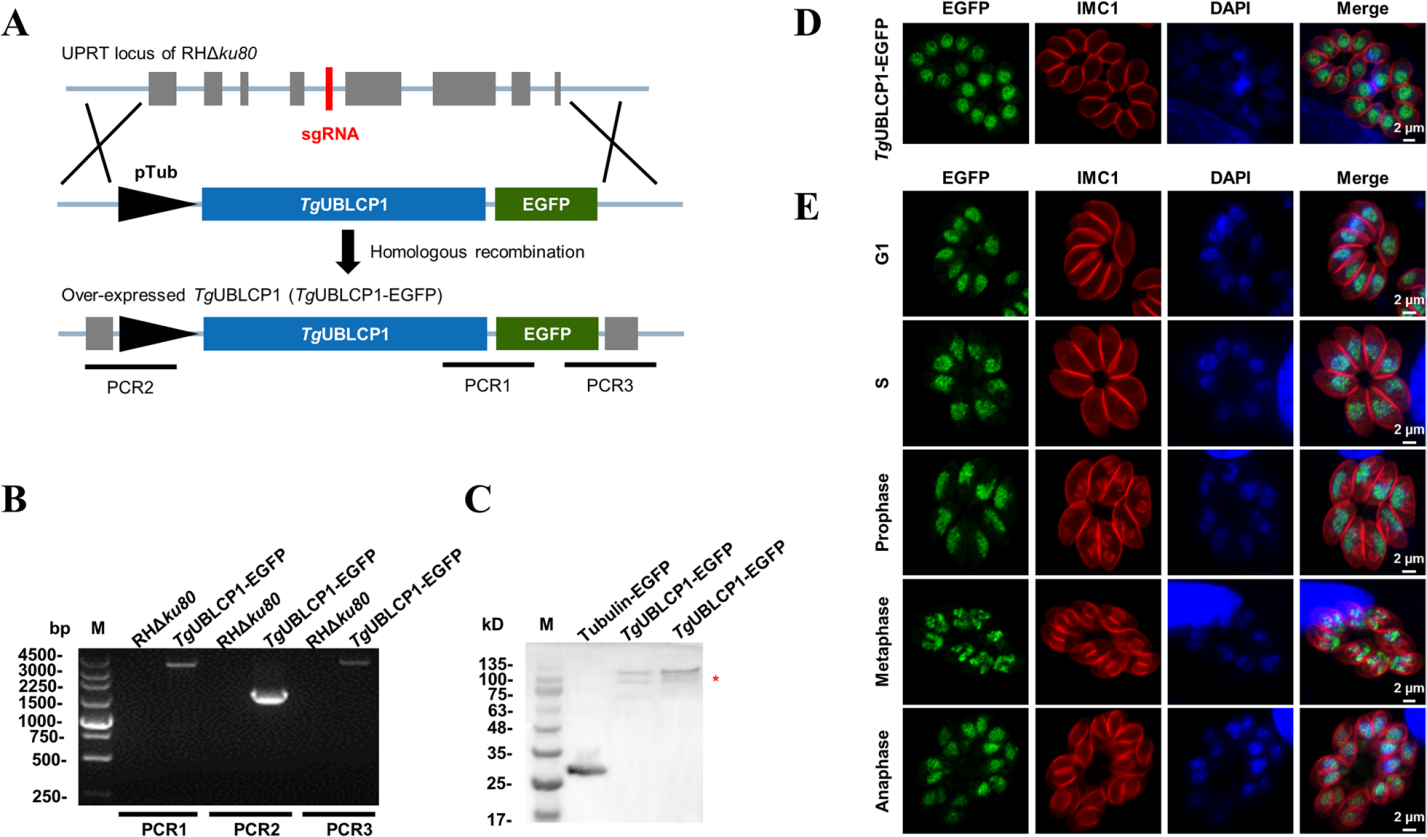
Characterization of UBLCP1 in *Toxoplasma gondii*. **A**, Diagram illustrating the CRISPR–Cas9-mediated homologous recombination of *T. gondii UBLCP1* at the uracil phosphoribosyl transferase (UPRT) site. The donor fragment is composed of full-length *UBLCP1* fused with the EGFP epitope, controlled by a tubulin promoter. **B**, PCR amplification analyses of target sequence (PCR1) and 5′ and 3′ integration of homologous fragments (PCR2 and PCR3) in UBLCP1-EGFP tachyzoites. **C**, Western blot analysis of UBLCP1-EGFP and Tub-EGFP strains with rabbit anti-GFP antibody. **D**, The subcellular localization of UBLCP1-EGFP in tachyzoites of *T. gondii*, as determined by indirect immunofluorescence. **E**, Subcellular localization of UBLCP1 is restricted to the nucleus at different stages of the *T. gondii* tachyzoite cell cycle. Green indicates rabbit anti-GFP antibody. Red indicates IMC1. Blue indicates DNA-specific dye with DAPI. Scale bar, 2 µm

Although the TGGT1_222010 was predicted to be a 26S proteasome phosphatase UBLCP1 coding gene, almost nothing is known about the role of this gene in *T. gondii*. We conducted an indirect immunofluorescence assay (IFA) to determine where the UBLCP1 localizes in *T. gondii*. Alternatively, using a CRISPR–Cas9-based method, we constructed a strain that overexpressed recombinant UBLCP1 under the drive of the pTubulin promoter (Fig. 1a–c), in which the subcellular localization of UBLCP1 was successfully indicated in the tachyzoites of *T. gondii* (Fig. 1d). UBLCP1 was detected predominantly in the nucleus and moderately in the cytoplasm, of *T. gondii* tachyzoites, at either the G1, S, pro-, meta-, or ana-phase of the cell cycle (Fig. 1e).

### Deletion of UBLCP1 compromises the lytic growth of *T. gondii*

**Fig. 2:**
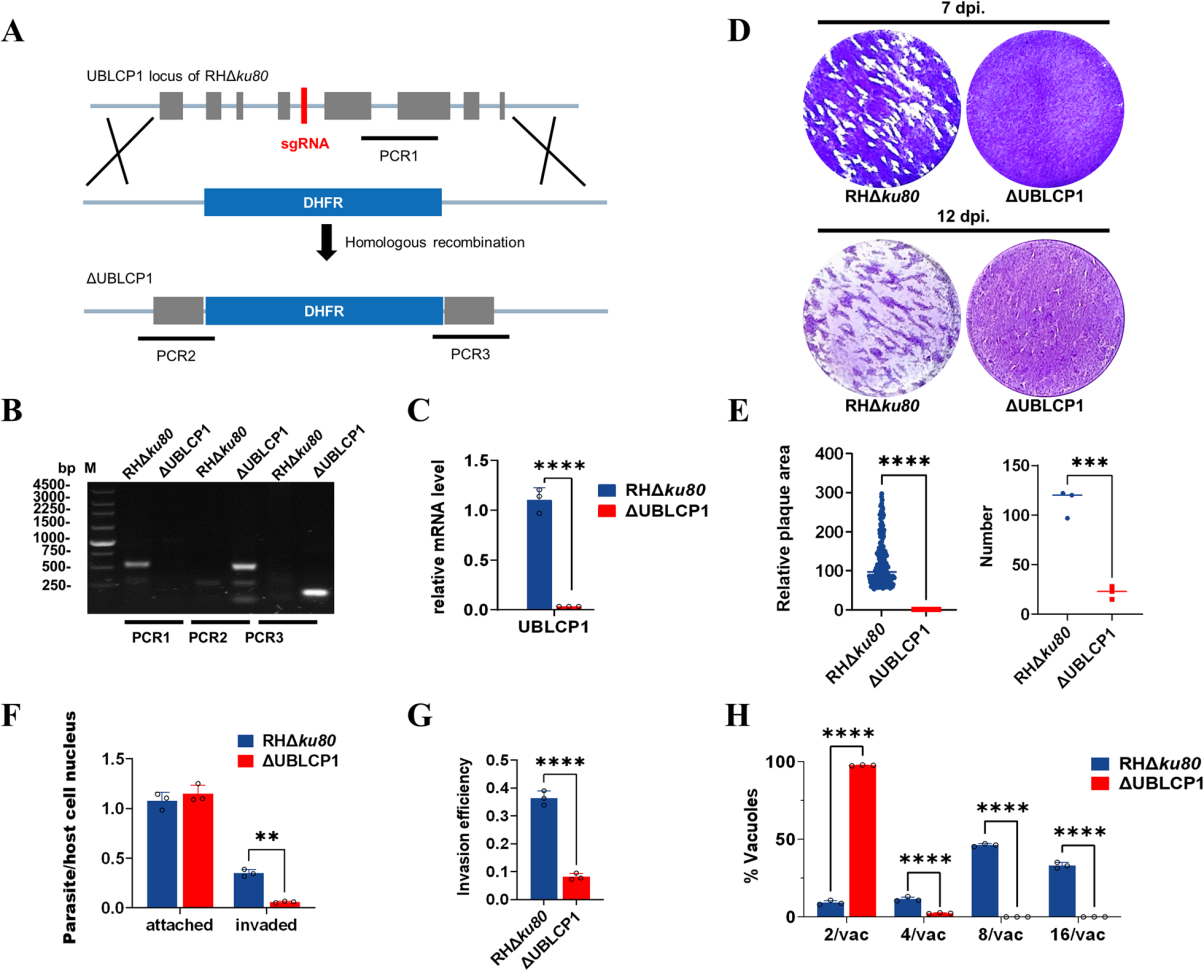
UBLCP1 is essential for parasite growth in *Toxoplasma gondii*. **A**, Schematic illustration showing the replacement of the coding sequence of UBLCP1 using a CRISPR–Cas9 method. **B**, PCR amplification of *UBLCP1* fragment in RHΔ*ku80* and mutant strains. PCR1 indicates the absence of UBLCP1; PCR2 and PCR3 indicate the 5′ and 3′ integration of homologous fragments, respectively. **C**, Relative mRNA levels of *UBLCP1* in the parental RHΔ*ku80* and ΔUBLCP1 strains of *T. gondii*. Data are presented as the mean ± standard deviation (SD) of three independent repeats. ****P ≤ 0.0001, using a *t*-test. **D**, Representative image of plaques observed in HFFs infected with the parental RHΔ*ku80* or ΔUBLCP1 strain 7 or 12 days postinfection. **E**, The relative number and area of plaques formed by tachyzoites of indicated strains 7 days postinfection. **F**, The number of attached and invaded parasites of RHΔ*ku80* or ΔUBLCP1 tachyzoites to/into HFFs. **G**, The invasion efficiency of RHΔ*ku80* and ΔUBLCP1 tachyzoites, presented as invaded parasites/total parasites. **H**, The intracellular replication rates of RHΔ*ku80* and ΔUBLCP1 are indicated by the number of tachyzoites per parasitophorous vacuole (PV). At least 100 vacuoles are analyzed for each mutant

To elucidate the biological significance of UBLCP1 to parasite fitness, we constructed a UBLCP1 deletion mutant strain of *T. gondii* (ΔUBLCP1), which was confirmed by PCR and qPCR (Fig. 2a–c). Deletion of UBLCP1 compromised the *T. gondii* growth over multiple lytic cycles in host cells. Plaques formed by tachyzoites were barely visible after 7 days and only became distinguishable at day 12 postinoculation. (Fig. 2d). ΔUBLCP1 resulted in significantly decreased number (*P* < 0.0001) and size (*P* < 0.0001) of plaques after 7 days in vitro compared with the parental RHΔ*ku80* (Fig. 2e). Deletion of UBLCP1 significantly reduced the number of tachyzoites in HFF cells (*P* < 0.01; Fig. 2f) and their overall invasion efficiency (*P* < 0.0001; Fig. 2g) compared with the parental RHΔ*ku80* strain. In the ΔUBLCP1-invaded HFF cells, more than 95% of parasitophorous vacuoles (PVs) contained only two tachyzoites after 24 h (Fig. 2h), showing significantly (*P* < 0.0001) compromised intracellular replication of the ΔUBLCP1 compared with the parental RHΔ*ku80*. These results show the significant involvement of UBLCP1 during the parasite replication of *T. gondii*.

### UBLCP1 is required for daughter budding and cell cycle progression of* T. gondii*

**Fig. 3:**
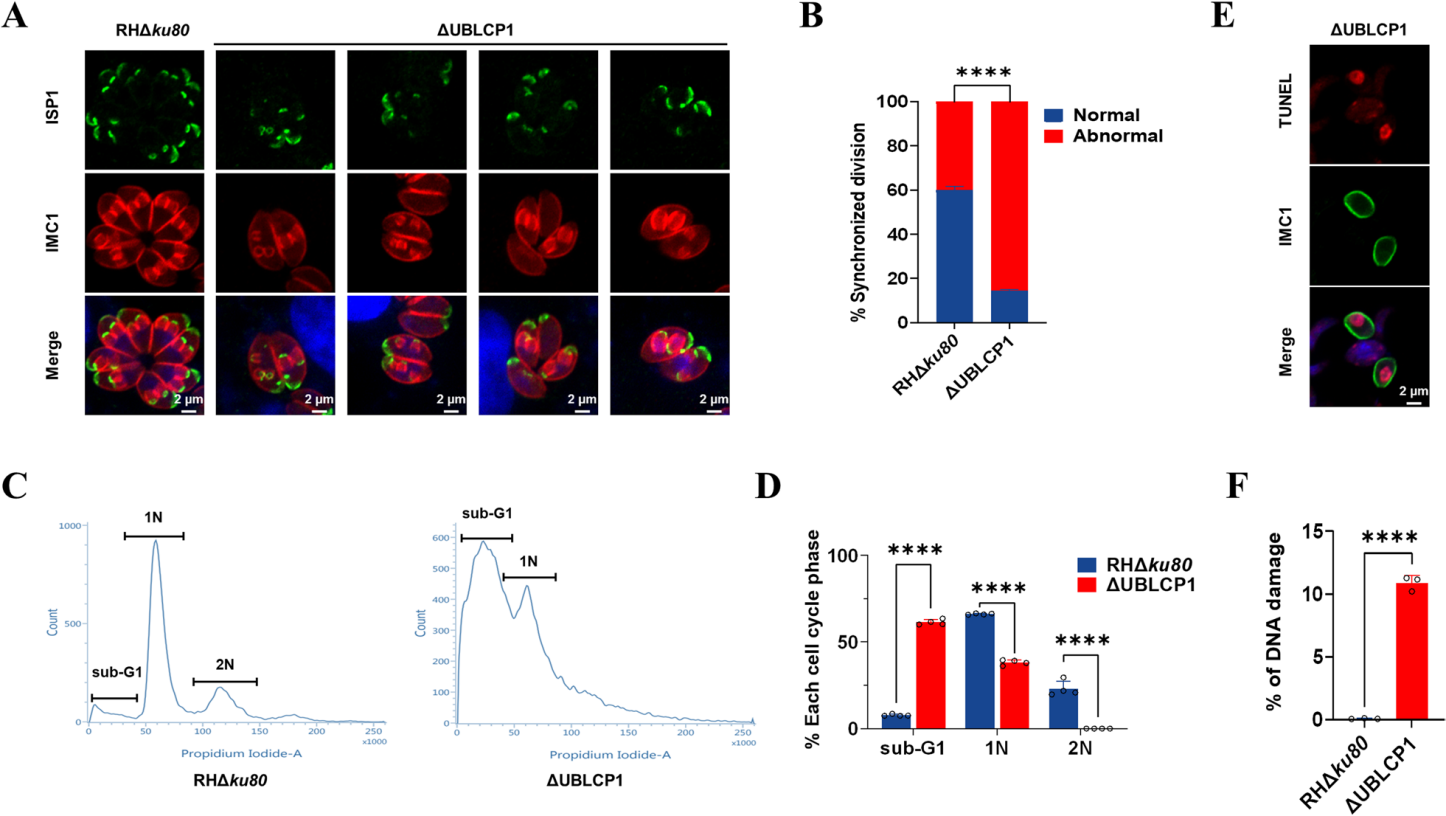
Disruption of UBLCP1 leads to asynchronous daughter budding and impairs the cell cycle of *Toxoplasma gondii*. **A**, Indirect immunofluorescence of ISP1 and IMC1 in RHΔ*ku80* and ΔUBLCP1 tachyzoites during cell division. Green indicates ISP1. Red indicates IMC1. Scale bar, 2 µm. **B**, The percentages of the asynchronous division of RHΔ*ku80* and ΔUBLCP1 tachyzoites. **C**, The DNA contents were assessed by propidium iodide (PI) staining to distinguish the cell cycle phase of *T. gondii*, for 1N: G1 phase; 1.2–1.8N: S/M phase; 2N: M/C phase. At least 10,000 PI-positive tachyzoites from each strain were analyzed by flow cytometry. **D**, Percentages of RHΔ*ku80* and ΔUBLCP1 tachyzoites subpopulation at sub-G1, 1N, and 2N phases. **E**, Representative image of a TUNEL-positive tachyzoite of ΔUBLCP1 strain with DNA damage. Red indicates TUNEL stain. Green indicates IMC1. Scale bar, 2 µm. **F**, DNA damage rates of RHΔ*ku80* and ΔUBLCP1 tachyzoites, as determined by flow cytometry after TUNEL staining. **P ≤ 0.01; ****P ≤ 0.0001; ***P ≤ 0.001, by unpaired *t* test

Tachyzoites of *T. gondii* rapidly proliferate by endodyogeny, a process in which two daughter cells develop internally within a parent [48]. As previously stated, UBLCP1 plays an indispensable role in the lytic growth of *T. gondii* tachyzoites, particularly during their replication stage. To further dissect what was causing the slower replication of the UBLCP1 deletion mutant, an endodyogeny event was observed for the ΔUBLCP1. Since most parasitophorous vacuoles (PVs) in the ΔUBLCP1 strain contained only two or four tachyzoites 24 h postinfection, we quantified asynchronous division events after culturing the parasites for 32 h [38]. With reference to the distribution of inner membrane complex (IMC) subcompartment protein 1 (ISP1, which is localized to the apical cap portion of the IMC), the asynchronous division was observed for ΔUBLCP1 tachyzoites (Fig. 3a). The asynchronous division rate of ΔUBLCP1 tachyzoites was quantified as 85.36 ± 0.36% compared with a 40% asynchronous division rate in the parental RHΔ*ku80* (Fig. 3b).

To investigate the cause of compromised lytic growth (e.g., host cell invasion and tachyzoite replication) in ΔUBLCP1, particularly its impact on asynchronous division, we compared the DNA content of ΔUBLCP1 and parental RHΔ*ku80* tachyzoites at the G1, M/C, and S/M phases (Fig. 3c). On the basis of the characteristics of the *T. gondii* haploid genome, the cell cycle of *T. gondii* was determined by comparing the DNA contents. For example, the DNA content of *T. gondii* tachyzoites in the G1 phase is defined as 1 N; meanwhile, the M/C phase contained double the DNA, represented as 2N, and during the S/M phase, which is in the replication phase, the DNA content ranges from 1.2N to 1.8N. Compared with RHΔ*ku80*, the percentage of the nonsynchronized culture of ΔUBLCP1 parasites at sub-G1 phase significantly (*P* < 0.0001) increased, whereas the percentage of ΔUBLCP1 at both the G0/G1 phase (DNA content defined as 1N; 38.18 ± 2.08%) and S/M phase (containing double DNA as 2N; 0.00%) significantly (*P* < 0.0001) decreased (Fig. 3d). As the sub-G1 phase usually indicates DNA damage in eukaryotic cells [49], we performed TUNEL staining for DNA damage analysis (Fig. 3e). About 10% of the ΔUBLCP1 tachyzoites exhibited DNA damage (Fig. 3f). These results suggest that UBLCP1 deletion caused DNA damage in a portion of parasites, which then arrested cell cycle progression and suppressed parasite replication.

### Deletion of UBLCP1 affects mitochondrial structure and function in *T. gondii*

**Fig. 4:**
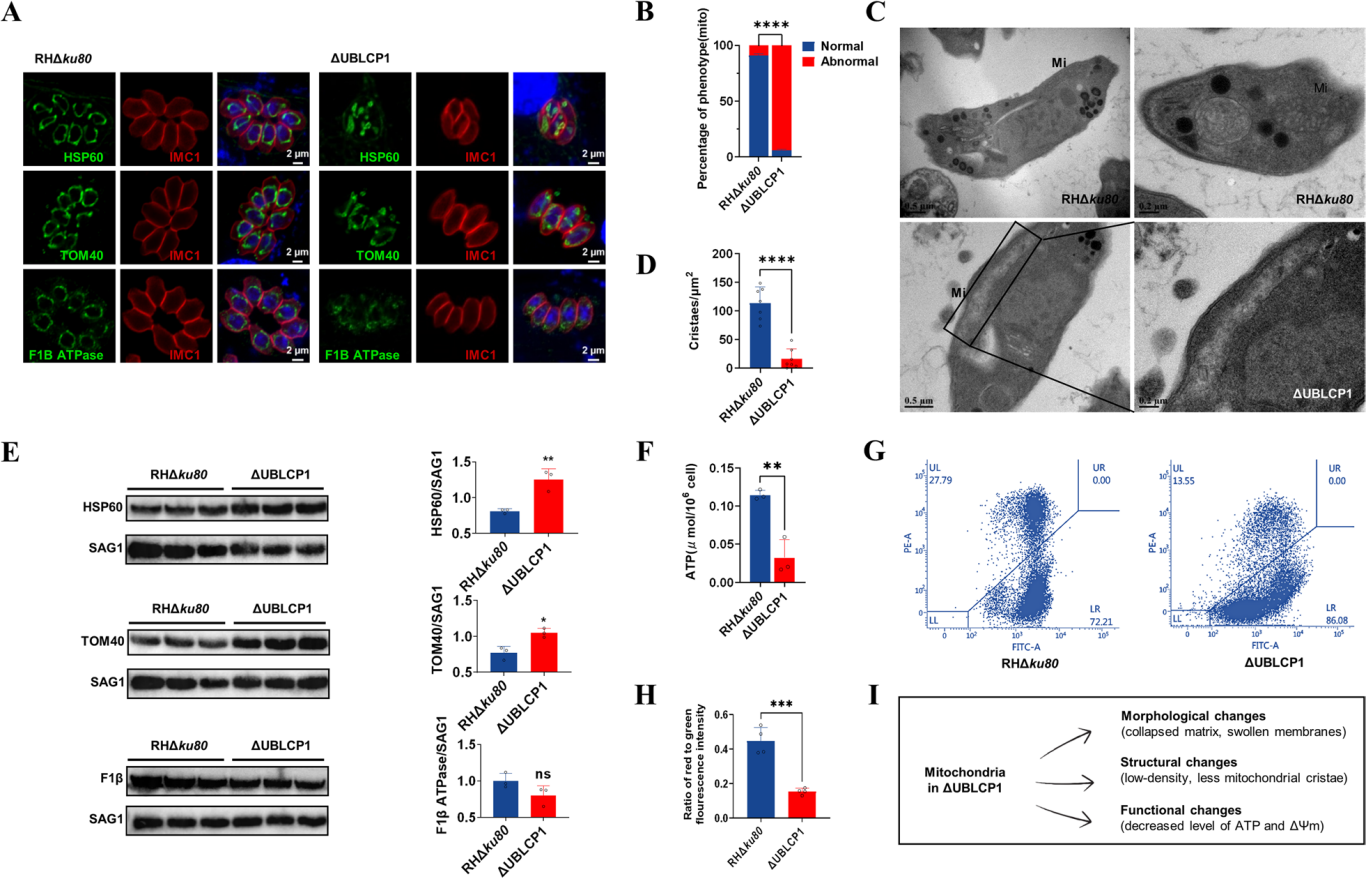
Mitochondrial morphology and structure are disrupted by the absence of UBLCP1 in *Toxoplasma gondii*. **A**, Indirect immunofluorescence of HSP60 (marker protein of mitochondrial matrix), TOM40 (marker protein of mitochondrial outer membrane), and F1β ATPase (marker protein of mitochondrial inner membrane) in RHΔ*ku80* and ΔUBLCP1 tachyzoites. Red indicates IMC1. Scale bar, 2 µm. **B**, Statistical analysis of collapsed mitochondrial morphology in RHΔ*ku80* and ΔUBLCP1 tachyzoites detecting by polyclonal rabbit anti-HSP60 sera. At least ten images for each sample were acquired stochastically with a Zeiss LSM880 confocal laser scanning microscope to analyze. **C**, Transmission electron micrographs of RHΔ*ku80* and ΔUBLCP1 tachyzoites. Insets show representative mitochondrion using Mi as an indication. The mitochondrion is outlined with black box lines and enlarged to show significantly reduced mitochondrial density and cristae number in ΔUBLCP1 tachyzoites. **D**, The number of mitochondrial cristae per μm^2^ of RHΔ*ku80* and ΔUBLCP1 tachyzoites. **E**, Protein expressions of HSP60, TOM40, and F1β ATPase in RHΔ*ku80* and ΔUBLCP1 tachyzoites, which are analyzed on the basis of the grey intensity of protein bands using ImageJ software. SAG1 is employed as the internal control. **F**, ATP content in RHΔ*ku80* and ΔUBLCP1 tachyzoites. **G**, Mitochondrial membrane potential (ΔΨm) of RHΔ*ku80* and ΔUBLCP1 tachyzoites, as assessed by JC-1 probe. Representative histograms of red fluorescence are recorded by flow cytometry. **H**, The ratio of red to green fluorescence intensity between RHΔ*ku80* and ΔUBLCP1 tachyzoites. Over 10,000 JC-1 positive tachyzoites of each strain are analyzed by flow cytometry. The values are the means ± standard deviation (SD). *P < 0.1; **P < 0.01, ***P ≤ 0.001, ****P ≤ 0.0001, by unpaired *t* test. **I**, The schematic diagram illustrates the mitochondrion abnormalities in terms of morphology, ultrastructure, and function in the UBLCP1 deletion strain

We examined organelle morphology using IFA and observed significant mitochondrial alterations in ΔUBLCP1 tachyzoites, consistent with previous findings (Supplementary Fig. S1). Then, to explain the alterations in mitochondrion following UBLCP1 deletion, we looked at the mitochondrial morphology, ultrastructure, expression level of mitochondrial marker proteins, and other aspects. Using a polyclonal antibody-based immunofluorescence assay with heat shock protein 60 (HSP60) as a mitochondrial matrix marker, we observed a collapsed, ball-like mitochondrial morphology in ΔUBLCP1 tachyzoites, contrasting with the typical lasso-shaped mitochondrion in RHΔ*ku80* tachyzoites (Fig. 4a). Using *T. gondii* TOM40 and F1β ATPase as the markers of mitochondrial outer and inner membranes, respectively, we showed that the outer membrane of mitochondrion was swollen (known as the “thick membranes” phenotype [23]), while the inner membrane was diffused in ΔUBLCP1 tachyzoites, compared with that of RHΔ*ku80* tachyzoites (Fig. 4a). The abnormal mitochondrial morphology ratio of the parental strain and ΔUBLCP1 tachyzoites was assessed by using polyclonal rabbit anti-HSP60 sera. The collapsed mitochondrial matrix was observed in > 90% PVs of ΔUBLCP1 tachyzoites (Fig. 4b). Transmission electron microscopy (TEM) revealed that mitochondria in ΔUBLCP1 tachyzoites had lower density and fewer cristae compared with those in RHΔ*ku80* tachyzoites (Fig. 4c, d). Such “thick membranes” and “collapsed mitochondrial” phenotypes were associated with increased protein levels of TOM40 and HSP60 (*P* < 0.1 and *P* < 0.01, respectively) and decreased protein level of F1β ATPase in ΔUBLCP1 tachyzoites, compared with that in RHΔ*ku80* tachyzoites (Fig. 4e). Next, we measured the mitochondrial membrane potential and the ATP content, which were key in influencing the functional state of mitochondrion. Compared with RHΔ*ku80*, significantly decreased levels of ATP (by approximately 70%; *P* < 0.01) and mitochondrial membrane potential (ΔΨm; *P* < 0.001) were detected in ΔUBLCP1 tachyzoites (Fig. 4f–h).

Reintroducing the *UBLCP1* gene into the UPRT locus of the ΔUBLCP1 strain generated a complementary strain (::UBLCP1), restoring both normal growth and plaque formation (Supplementary Fig. S2). Defective mitochondrial integrity and morphology were completely rescued in the ::UBLCP1 tachyzoites (Supplementary Fig. S2). These results suggest a role of UBLCP1 in the mitochondrial biology (e.g., morphology, ultrastructure, and function) of *T. gondii* (Fig. 4i).

### Deletion of UBLCP1 alters the phosphorylation levels of multiple proteins involved in cell cycle progression and division in* T. gondii*

**Fig. 5:**
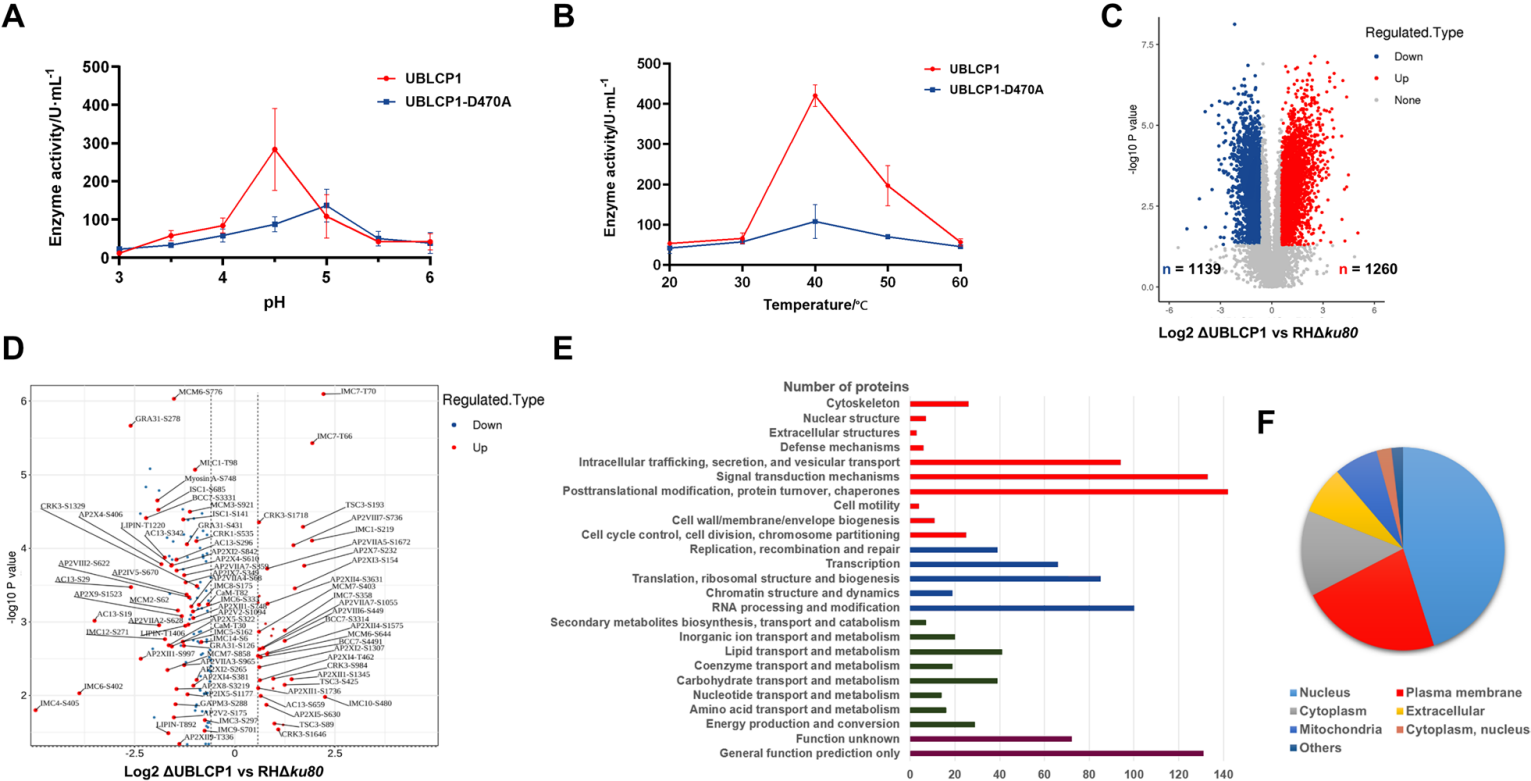
Functional annotation of the phosphatase UBLCP1. **A**, **B**, The phosphatase activity of UBLCP1 in the optimum reaction pH (**A**) or the optimum reaction temperature (**B**). **C**, Volcano plot showing the alteration of phosphorylation proteins between RHΔ*ku80* and ΔUBLCP1 tachyzoites. The upregulated phosphorylation of proteins with fold-changes ≥ 1.5 and *P* < 0.05 and downregulated phosphorylation of proteins with fold changes ≤ −1.5 and *P* < 0.05 are represented in red and blue, respectively. **d,** Volcano plot showing the differentially phosphorylated proteins related to chromosome organization and duplication; cell cycle progression and cell division have more than one significant differentially phosphorylated site. **e**, Functional enrichment of differential phosphoproteins between RHΔ*ku80* and ΔUBLCP1 tachyzoites in cellular processes and signaling (red), information storage and processing (blue), metabolism (green) and poorly characterized (black) categories. **f**, Pie chart of cellular component annotation categories for differential phosphoproteins between RHΔ*ku80* and ΔUBLCP1 tachyzoites

To obtain more information on the molecular function of UBLCP1 and the biological processes it involves, the phosphatase activity of UBLCP1 was first determined in vitro. UBLCP1 is an acid phosphatase with an optimum reaction pH of about 4.5 (Fig. 5a). Moreover, the optimum reaction temperature was about 40 ℃ (Fig. 5b). Next, we performed a comparative phosphoproteomic study between the ΔUBLCP1 and RHΔ*ku80*. With reference to RHΔ*ku80*, a total of 1,260 phosphoproteins were upregulated (fold-change of > 1.5; *P* < 0.05), and 1,139 phosphoproteins were downregulated (fold-change of < −1.5; *P* < 0.05) in ΔUBLCP1 (Fig. 5c; Table S1). Related to the progress of the *T. gondii* tachyzoites cell cycle and division, AP2 domain transcription factors, inner membrane complex (IMC) proteins, and IMC-related proteins were conspicuously identified, some of which were detected with more than one differentially phosphorylated site (Fig. 5d; Supplementary Table S1). The differential phosphoproteins were predicted to be functionally enriched in biological processes such as posttranslational modification, protein turnover, chaperones, signal transduction mechanisms, intracellular trafficking, secretion, and vesicular transport (Fig. 5e). Most of the differentially phosphorylated proteins were predicted to localize in the nucleus (877/1945), plasma membrane (434/1945), and cytoplasm (266/1945); of note, the mitochondrion (137/1945) was the organelle enriched with part of differentially phosphorylated proteins (Fig. 5f).

### UBLCP1 is crucial for *T. gondii* tachyzoite proliferation in vivo

**Fig. 6:**
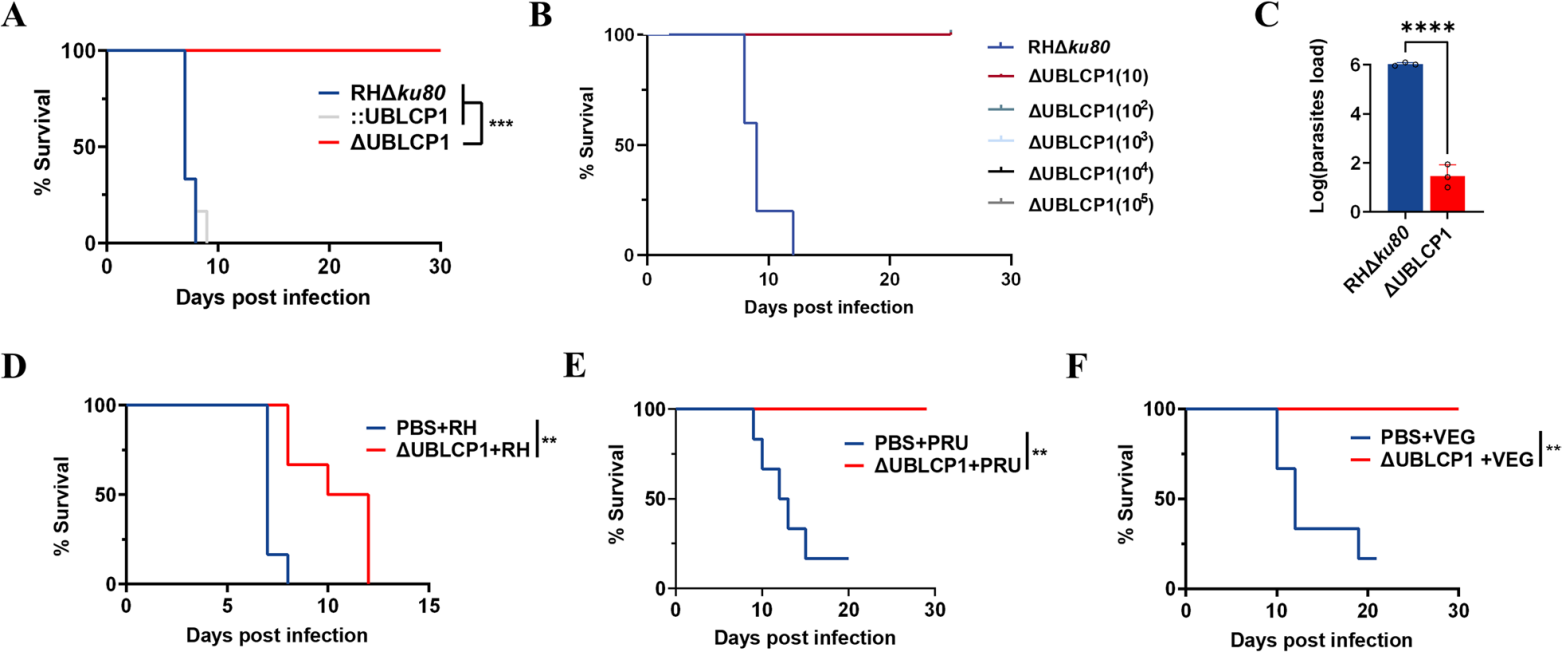
UBLCP1 is necessary for the virulence of *Toxoplasma gondii*. **A**, Survival of ICR mice injected intraperitoneally with 100 tachyzoites of RHΔ*ku80*, ΔUBLCP1 or::UBLCP1 strain. **B**, Survival of ICR mice injected intraperitoneally with different quantities of ΔUBLCP1 tachyzoites. **C**, The proliferation of RHΔ*ku80* or ΔUBLCP1 tachyzoites in ICR mice peritoneal fluids determined by quantitative PCR 5 days after intraperitoneal infection with 100 tachyzoites. **D**–**F**, The efficiency of immune protection of ICR mice vaccinated with 10^4^ ΔUBLCP1 tachyzoites from challenge with 10^4^ tachyzoites of type I strain RH (**D**), type II strain PRU (**E**), or type III strain VEG (**F**) of *T. gondii* 30 days postimmunization, and the survival of mice is monitored for another 30 days. **P ≤ 0.01; ***P ≤ 0.001; ****P ≤ 0.0001; by unpaired *t*-test

Stimulated by the results of UBLCP1 and its crucial roles in the lytic cycle and mitochondrial integrity of *T. gondii* tachyzoites, we tested their essentialities in parasite infection and pathogenesis in vitro. Notably, mice infected with 100 RHΔ*ku80* or ::UBLCP1 tachyzoites succumbed within 2 weeks, whereas those infected with ΔUBLCP1 tachyzoites survived without clinical signs (Fig. 6a). Mice also survived from the infection with 1,000, 10,000, and even 100,000 ΔUBLCP1 tachyzoites, suggesting attenuated virulence of the UBLCP1 deletion mutant (Fig. 6b). Specifically, at 5 days postinfection, the parasite burden of RHΔ*ku80* in mouse peritoneal fluids was determined, which increased from 100 tachyzoites to about 1,000,000 tachyzoites, whereas the parasite burden of ΔUBLCP1 strain did not increase (Fig. 6c), suggesting a deficiency of parasite replication in vivo.

Given the crucial role of UBLCP1 and its related proteins in the proliferation and pathogenicity of *T. gondii*, we expanded our findings to the potential of UBLCP1 as a vaccine candidate. Potential immune protection elicited by ΔUBLCP1 vaccination in mice was evaluated. Compared with negative controls, mice inoculated with 10,000 ΔUBLCP1 tachyzoites showed an extended survival time when challenged with 10,000 tachyzoites of the type I strain RH of *T. gondii* (Fig. 6d) and survived a challenge with 10,000 tachyzoites of the type II strain PRU or type III strain VEG of *T. gondii* (Fig. 6e, f).

## Discussion

Recently, there have been major findings regarding *T. gondii* protein phosphatases, which mediate either the attachment, invasion, replication, nutrient acquisition, tachyzoite-bradyzoite differentiation or immune evasion to affect the virulence of *T. gondii* infection [28, 38, 40, 50–54]. In this study, we demonstrated that the phosphatase UBLCP1 is associated with mitochondrial integrity and required for lytic growth, cell cycle progression, and virulence in the *T. gondii* parasite.

UBLCP1 is a haloacid dehalogenase (HAD)-like phosphatase of the F-cell production 1/small CTD (carboxy-terminal domain, RNA polymerase II, polypeptide A) phosphatase (FCP/SCP) family of protein serine/threonine phosphatases (PSPs) [43, 55]. Owing to the extremely low abundance of UBLCP1 according to the information from ToxoDB (https://toxodb.org), we failed to localize this protein in *T. gondii* tachyzoites by performing either endogenous labeling or polyclonal antibodies-based experiments (Supplementary Figs. S3–S4). Although the canonical nuclear localization signal was not predicted, the UBL domain of this phosphatase has been reported as sufficient for nuclear localization [24]. By constructing an overexpressing strain, although it might introduce false positive results, we eventually found a localization of UBLCP1 in the nucleus and cytoplasm of tachyzoite, which is consistent with other eukaryotes [56].

Although UBLCP1 is designated a dispensable protein with a mild fitness score (phenotype score = −0.63) in the genome-wide CRISPR screen [57], in the current study, phenotypes (e.g., compromised lytic growth, compromised intracellular replication, reduced invasion ability, and defect invasion efficiency) are observed in the UBLCP1 deletion tachyzoites of *T. gondii*. Furthermore, to distinguish whether UBLCP1 affects motility or invasion mechanisms, more experiments, such as gliding motility assay, can be performed to comprehensively explore the role of UBLCP1 in *T. gondii* growth over multiple lytic cycles in host cells.

In this study, the deletion of UBLCP1 led to compromised proliferation of *T. gondii* tachyzoites in cells. Tachyzoite proliferation begins with daughter budding [13, 58], and multiple protein phosphatases, such as PP6C, PPKL, and haloacid dehalogenase phosphatase HAD2a in *T. gondii*, have been reported as essential for the parasite division and daughter parasite maturation [38, 53, 54]. We found that tachyzoites lacking UBLCP1 exhibited asynchronous division and abnormal formation of daughter buds within PVs of the parental parasite (ΔUBLCP1), suggesting a role of UBLCP1 in the cell cycle and division of the asexual, acute infection stage of *T. gondii*. This statement is supported by the phosphoproteomic data, which revealed differential phosphorylation of proteins including AP2 domain transcription factors, IMCs, and IMC component proteins, cyclin-dependent kinase-related kinases CRK1 and CRK3, cyclin PHO80, chromosome structure modulators RCCs, and mini-chromosome maintenance proteins [59–63]. These proteins were predicted to be involved in the regulation of chromosome organization and duplication, cell cycle progression, and cell division [64, 65], and they were significantly upregulated in ΔUBLCP1 compared with that in the parental strain. Disruption of UBLCP1 also altered the phosphorylation status of calcium-dependent protein kinase proteins (CDPKs), which have been demonstrated to make sense of invasion, motility, and egress of *T. gondii* tachyzoites [66, 67]. Notably, most of the differentially phosphorylated proteins were enriched in biological processes including posttranslational modification, protein turnover, chaperones, signal transduction mechanisms, intracellular trafficking, secretion, and vesicular transport.

Furthermore, the phosphatases of the FCP/SCP family possess a conserved motif DxDxT/V, which mutation of the first aspartic acid in DxDxT/V (shown in bold) lead to loss of phosphatase activity [43, 55]. The motif ^470^DLDYT^474^ is found in UBLCP1. We purified recombinant protein UBLCP1 and mutant protein UBLCP1-D470A for the determination of protein serine/threonine phosphatase activity (Supplementary Fig. S5). These results showed that this motif is the catalytic active site of UBLCP1, and UBLCP1 exhibited an optimum reaction pH value at 4.5 and an optimum temperature at 45 ℃. In addition, the optimal reaction pH value of UBLCP1 phosphatase activity was similar to human UBLCP1, further supporting our findings.

The mitochondrion plays an indispensable role in *T. gondii* morphology, structure, and coordination of daughter cell formation and organelle biogenesis [4, 12, 13]. Disruption of mitochondrial proteins typically leads to aberrant mitochondrial phenotypes, such as interconnected mitochondrion, mitochondrion with swollen membranes, “ball-like” or collapsed mitochondrial phenotype, aberrant mitochondrial cristae ultrastructure, and disruption of the mitochondrial membrane potential [23, 68, 69]. In the current study, it was found that the deletion of phosphatase UBLCP1 resulted in a collapsed mitochondrial matrix, swollen mitochondrial outer membrane, and diffusely distributed inner membrane. Particularly, the disruption of UBLCP1 resulted in a loss of mitochondrion density, reduced number of mitochondrial cristae, insufficient membrane potential, and ATP production. Although further experiments should be conducted to verify the role of UBLCP1 in mitochondrial biology, there is a relationship of this protein with the morphology and function of the mitochondrion in *T. gondii* tachyzoites.

Considering the crucial roles of UBLCP1 in parasite propagation and virulence in vivo, we explored the possibility of using UBLCP1 as a live-attenuated vaccine strain to prevent acute *Toxoplasma* infection. It was shown that ΔUBLCP1 immunization stimulated efficient protection against the challenge of virulent *T. gondii* strains. Although the work from mouse models demonstrated the great potential of this mutant as a vaccine candidate, to strengthen the rationale, additional studies should be conducted to further explore the memory immune responses (e.g., CD4^+^/CD8^+^ T cells and cytokines) postvaccination, challenge with additional *Toxoplasma* strains, or evaluate long-term protection beyond 30 days.

Apart from the exciting findings, some limitations and issues need to be further addressed for a better understanding of the role of UBLCP1 in *T. gondii*. First, a conditionally regulatable system is preferably employed to further explore the roles of UBLCP1 in *T. gondii*, particularly in aspects of mitochondrial integrity, lytic cycle, and virulence, and in answering the question of whether the mitochondrial phenotype is directly caused by the loss of UBLCP1. Second, it is necessary to include the complemented line in all phenotypic analyses in further exploration. In this study, we compared the plaque assay experiments of the wild type, deficient strain, and complementary strain to determine that UBLCP1 supplemented restored the plaque formation ability. Furthermore, the morphology of mitochondrion in the UBLCP1 complementary strain was restored to normal. Third, a comprehensive bioinformatic mining of the comparative phosphoproteomic dataset for ΔUBLCP1 and the control strain of *T. gondii* is needed to provide more clues for the specific involvements of UBLCP1 in molecular function, signaling pathway, and biological processing. A better understanding of these aspects should underpin the biological research and vaccine target discovery for *T. gondii* parasite.

## Conclusions

Taken together, we identify a phosphatase UBLCP1 that is associated with mitochondrial integrity and required for the lytic cycle in *T. gondii* tachyzoites. The deletion mutant of UBLCP1 shows compromised proliferation and virulence but retains the ability to elicit sufficient immune protection against the acute infection of this parasite. Although there is a possible link between the UBLCP1 mutant and the mitochondrial integrity in *T. gondii*, and the UBLCP1 is a vaccine/drug target candidate for the control of toxoplasmosis in animals, the specific involvements and detailed mechanisms of UBLCP1 in mitochondrial integrity and health remain to be further elucidated.

## Supplementary Information

Additional file 1: Figure S1. Effects of deficiency UBLCP1 on the organelle morphology in *Toxoplasma gondii*. IMC1 (green) and DAPI (blue) stains are used to denote the parasites and nuclei (DNA), respectively. Anti-SERCA, anti-CPN60, anti-Sortilin and anti-HSP60 antibodies are used to stain the endoplasmic reticulum, apicoplast, Golgi and mitochondrion, respectively. Scale bar: 2 μm.

Additional file 2: Figure S2. Construction and functional characterization of the UBLCP1 complemented strain of *Toxoplasma gondii*. (a) Diagram illustrating the CRISPR–Cas9-mediated UBLCP1 gene complement by replacing the UPRT gene locus with a UBLCP1-EGFP expression cassette in ΔUBLCP1 strain. (b) PCR amplification analysis of the presence of UBLCP1 (PCR1), the integration of UBLCP1-EGFP expression cassette (PCR2) in the parental RHΔ*ku80*, ΔUBLCP1 and ::UBLCP1 strain tachyzoites. (c) Immunofluorescence staining of UBLCP1-EGFP (green) in the ::UBLCP1 strains, with IMC1 (red) as a control. The blue, DNA-specific dye with DAPI. Scale bar, 2 µm. (d) Plaque assay of indicated strains cultured after 7 days. (e) The relative number and area of plaques formed by tachyzoites of indicated strains are statistically calculated and data is graphed as scatter diagrams. ****, *P* ≤ 0.0001; ***, *P* ≤ 0.001; *, *P* ≤ 0.1, all by unpaired *t* tests. (f) Mitochondrial morphology indicated in the parental RHΔ*ku80*, ΔUBLCP1 and ::UBLCP1 strains tachyzoites by immunofluorescence staining. Green indicates HSP60; red indicates IMC1; blue indicates DNA-specific dye by DAPI. Scale bar, 2 µm.

Additional file 3: Figure S3. Endogenous epitope tagging of UBLCP1 holoenzyme in *Toxoplasma gondii*. (a) Schematic diagram showing the strategy for 6HA endogenous epitope tagging to UBLCP1 at the C-terminus, with hypoxanthine xanthine guanosine phosphoribosyl transferase (HXGPRT) resistance cassette incorporated for the selection using mycophenolic acid and xanthine. (b) PCR amplification of integrated UBLCP1-6HA in *T. gondii*. (c) The subcellular localization of UBLCP1 in tachyzoites is indicated based on endogenous tagging by indirect immunofluorescence. Green indicates rabbit anti-HA antibody. Red indicates IMC1. Blue indicates DNA-specific dye with DAPI. Scale bar, 2 µm. (d) Western blot analysis of UBLCP1 in RHΔ*ku80* and UBLCP1-6HA strains, and blots are probed with rabbit anti-HA antibody to visualize target bands.

Additional file 4: Figure S4. Localization of UBLCP1 in *Toxoplasma gondii* using mouse anti-UBLCP1 polyclonal antibodies. (a) PCR amplification of truncated fragment UBLCP1, which is ligated into the pET-32a(+) expression vector via the *Bam*H I and *Hind* III restriction enzyme sites for protein expression in bacteria *E. coli* BL21 (DE3). (b) Prokaryotic expression of recombinant protein UBLCP1 (rUBLCP1) and SDS-PAGE. The optimal conditions for protein expression are 0.2 mM IPTG at 16°C for 12 h. M: Marker; Lanes 1, 2, 3, 4, 5 represent samples with different induction hours 0, 2, 4, 6, and 8 h, respectively; sup: supernatant; pre: precipitate. (c) Purification of recombinant UBLCP1. A gradient concentration of imidazole is used as an eluent. M: Marker; Lanes 1, 2, and 3 represent samples purified by 60 mM imidazole; Lanes 3-14 represent samples elucidated by 250 mM imidazole. (d) Western blot analysis of recombinant UBLCP1. Mouse anti-His antibody (1:1000) and goat anti-mouse IgG HRP-conjugated antibody (1:2000) are used as the primary and secondary antibodies, respectively. Lane 1: recombinant pET-32a protein as a control; Lane 2: recombinant UBLCP1. (e) Diagram illustrating the immunization process of mice using the purified recombinant UBLCP1. (f) The subcellular localization of UBLCP1 in *T. gondii *tachyzoites. Green indicates mouse anti-UBLCP1 polyclonal antibody. Red indicates IMC1. Blue indicates DNA-specific dye by DAPI. Scale bar, 2 µm. (g) Western blot analysis of recombinant pET32a protein, recombinant UBLCP1 and RHΔ*ku80*, blots are probed with mouse anti-UBLCP1 polyclonal antibodies.

Additional file 5: Figure S5. Determination of UBLCP1 phosphatase activity in vitro. (a) and (b) Induced expression, expression characteristic analysis and purification of recombinant protein UBLCP1 (a) or the mutation protein UBLCP1-D470A (b). The left panels show the induced expression of recombinant protein UBLCP1 or the mutation protein UBLCP1-D470A; M: Marker; 1, 2, 3, 4, 5 represent samples with different induction hours 0/2/4/6/8 h respectively. The middle panels show the obvious bands exclusively both in the insoluble fraction and the soluble fraction determined by SDS-PAGE results; sup: supernatant, pre: precipitate. The right panel shows the purification of UBLCP1 or UBLCP1-D470A protein using the elution buffer (25 mM tris-HCl, 300 mM NaCl, and 250 mM imidazole). M: Marker; 1–14 represent samples purified by the elution buffer. (c) Purified recombinant proteins identification by western blot using mouse anti-His antibody (1:1000) and goat anti-mouse IgG HRP-conjugated antibody (1:2,000). (d) Drawing of phosphate standard curve. The absorbance of standard solutions (phosphate content at 100, 200, 500, 1000 and 2000 pmol·(50 μL)^-1^) at 600 nm is determined spectrophotometrically using the molybdate dye method. In vitro phosphatase assay is carried out according to the manufacturer’s suggestion (Promega, USA).

Additional file 6: Table S1. Summary of the phosphor-proteomic analysis of ΔUBLCP1 and wild-type strain.

Additional file 7: Table S2. Primers used in this study.

## Data Availability

No datasets were generated or analyzed during the current study.
