# Supplementary figures and images for: Phosphatase UBLCP1 is required for the growth, virulence and mitochondrial integrity of Toxoplasma gondii
Source: Parasit Vectors. 2025 Mar 28;18:122. doi: 10.1186/s13071-025-06766-3 (PMC11951701; doi:10.1186/s13071-025-06766-3)

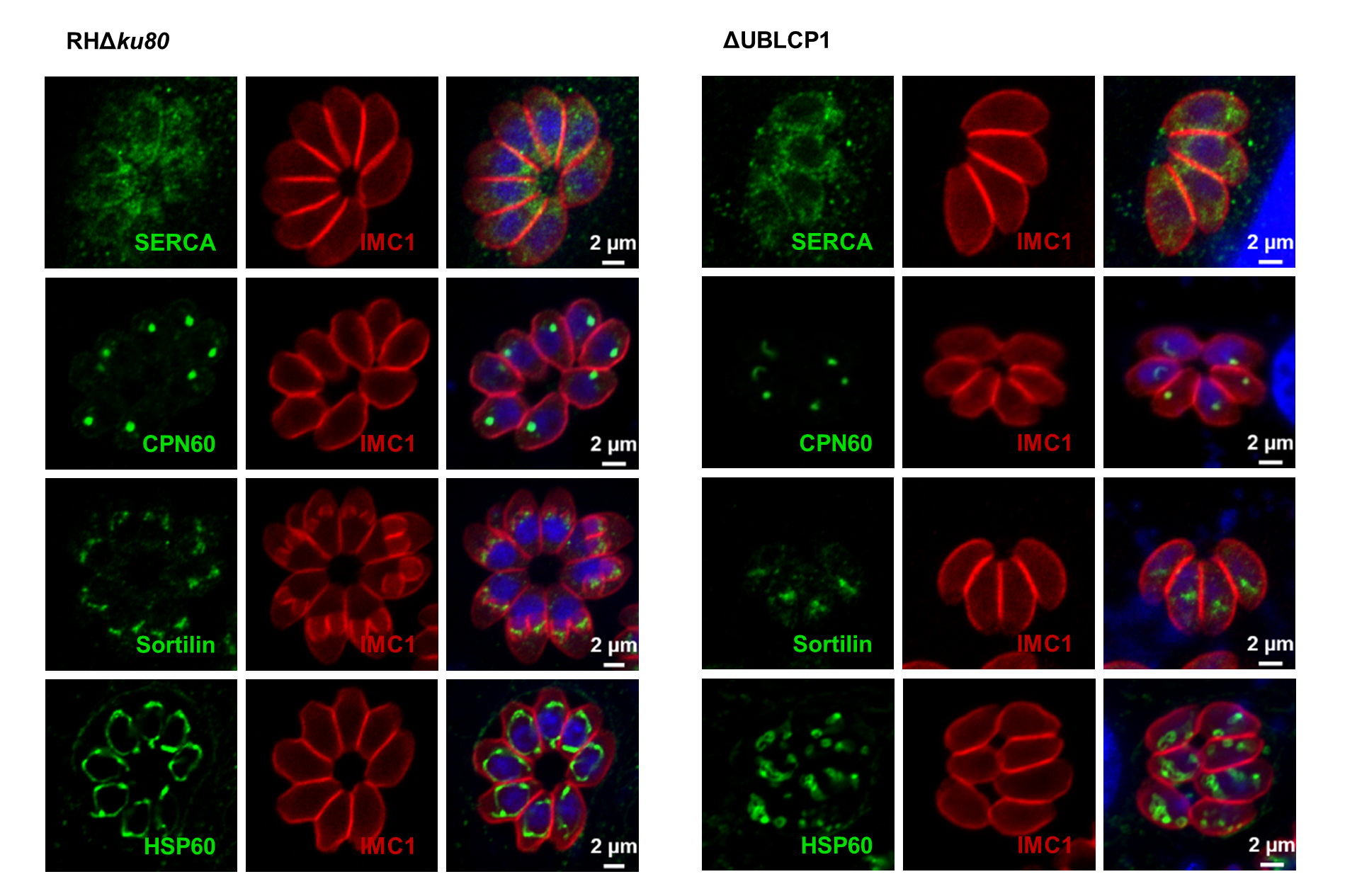

Supplement: Supplementary file 1 — Additional file 1: Figure S1. Effects of deficiency UBLCP1 on the organelle morphology in Toxoplasma gondii. IMC1 (green) and DAPI (blue) stains are used to denote the parasites and nuclei (DNA), respectively. Anti-SERCA, anti-CPN60, anti-Sortilin and anti-HSP60 antibodies are used to stain the endoplasmic reticulum, apicoplast, Golgi and mitochondrion, respectively. Scale bar: 2 μm. [file 13071_2025_6766_MOESM1_ESM.tif]

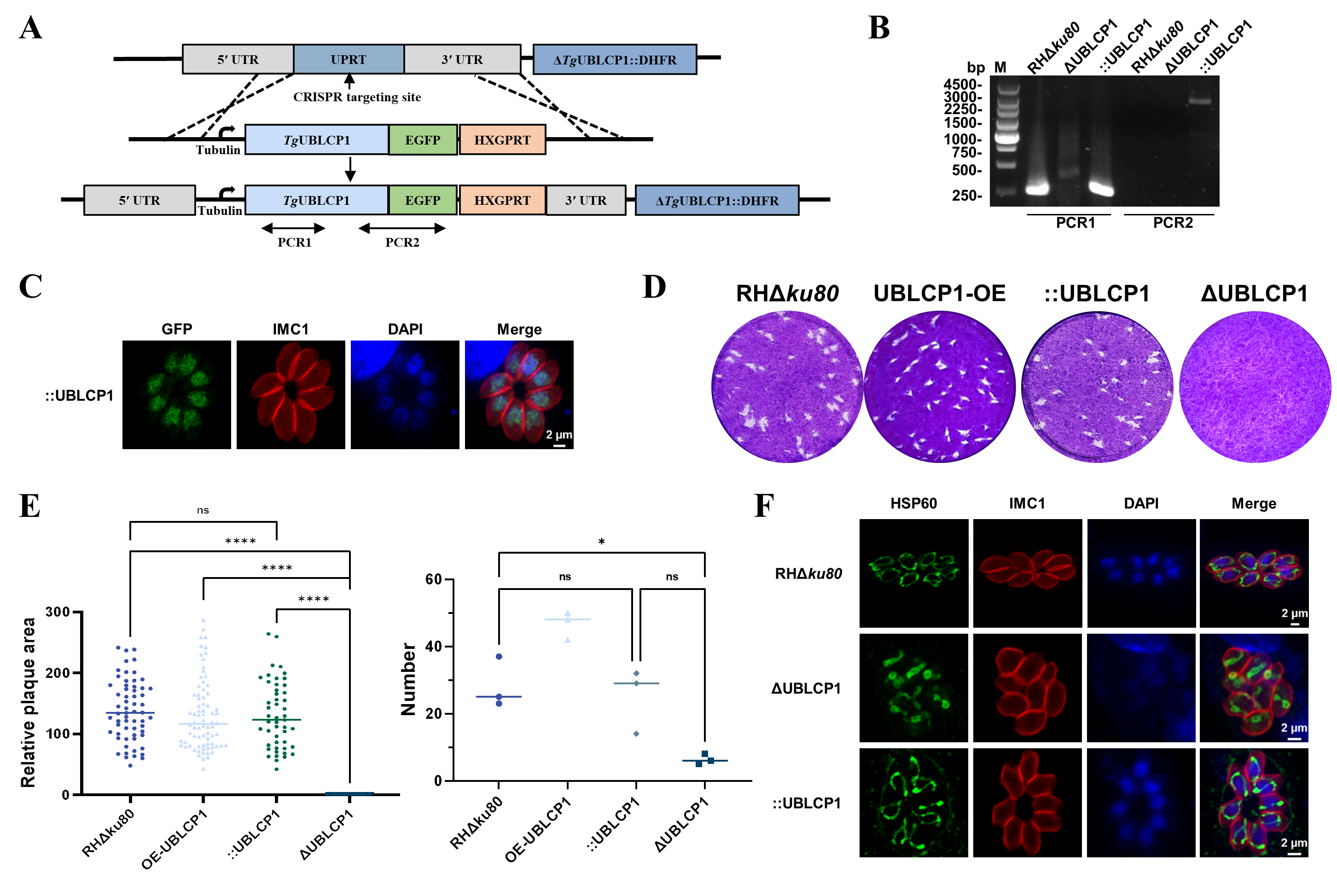

Supplement: Supplementary file 2 — Additional file 2: Figure S2. Construction and functional characterization of the UBLCP1 complemented strain of Toxoplasma gondii. (a) Diagram illustrating the CRISPR–Cas9-mediated UBLCP1 gene complement by replacing the UPRT gene locus with a UBLCP1-EGFP expression cassette in ΔUBLCP1 strain. (b) PCR amplification analysis of the presence of UBLCP1 (PCR1), the integration of UBLCP1-EGFP expression cassette (PCR2) in the parental RHΔku80, ΔUBLCP1 and ::UBLCP1 strain tachyzoites. (c) Immunofluorescence staining of UBLCP1-EGFP (green) in the ::UBLCP1 strains, with IMC1 (red) as a control. The blue, DNA-specific dye with DAPI. Scale bar, 2 µm. (d) Plaque assay of indicated strains cultured after 7 days. (e) The relative number and area of plaques formed by tachyzoites of indicated strains are statistically calculated and data is graphed as scatter diagrams. ****, P ≤ 0.0001; ***, P ≤ 0.001; *, P ≤ 0.1, all by unpaired t tests. (f) Mitochondrial morphology indicated in the parental RHΔku80, ΔUBLCP1 and ::UBLCP1 strains tachyzoites by immunofluorescence staining. Green indicates HSP60; red indicates IMC1; blue indicates DNA-specific dye by DAPI. Scale bar, 2 µm. [file 13071_2025_6766_MOESM2_ESM.tif]

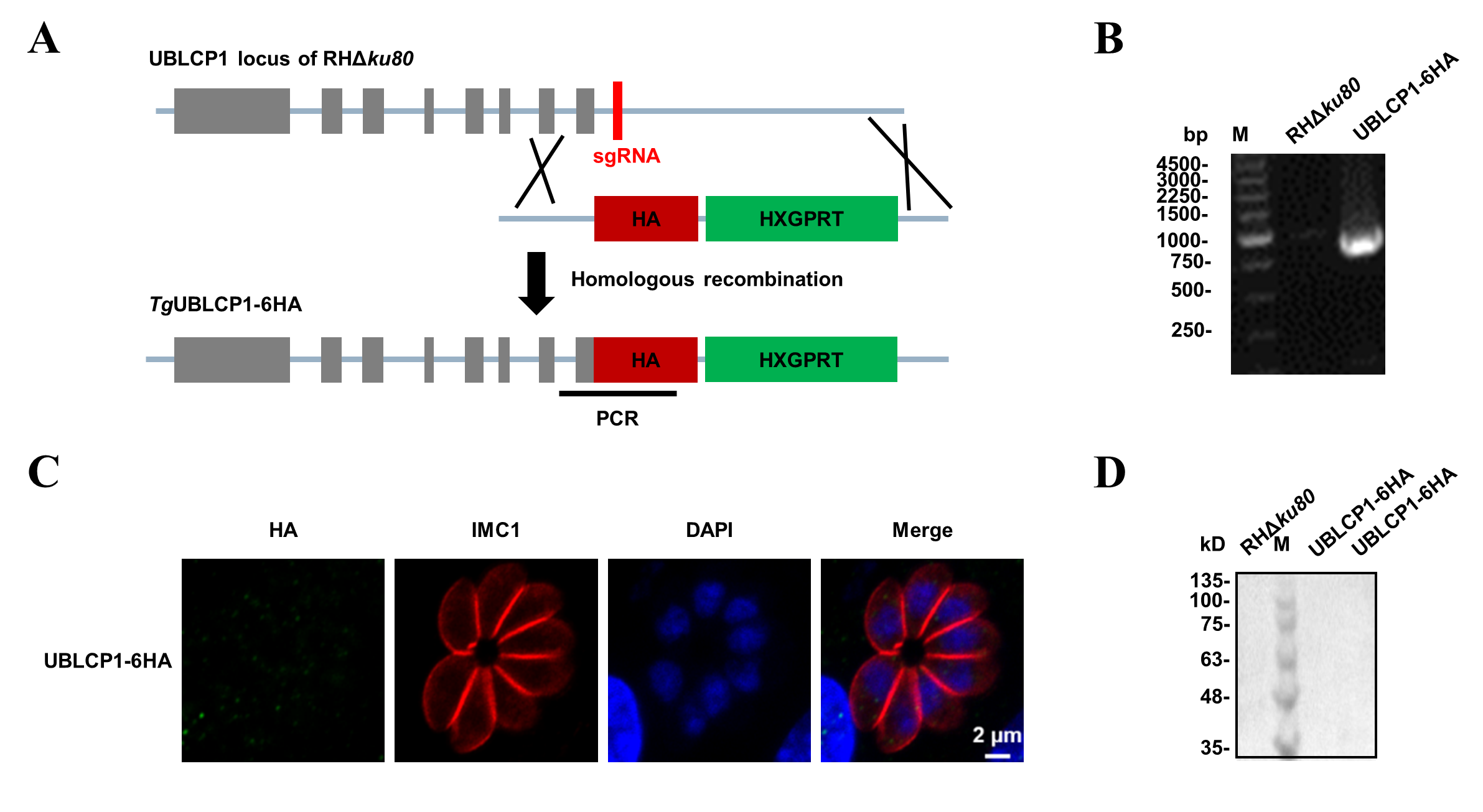

Supplement: Supplementary file 3 — Additional file 3: Figure S3. Endogenous epitope tagging of UBLCP1 holoenzyme in Toxoplasma gondii. (a) Schematic diagram showing the strategy for 6HA endogenous epitope tagging to UBLCP1 at the C-terminus, with hypoxanthine xanthine guanosine phosphoribosyl transferase (HXGPRT) resistance cassette incorporated for the selection using mycophenolic acid and xanthine. (b) PCR amplification of integrated UBLCP1-6HA in T. gondii. (c) The subcellular localization of UBLCP1 in tachyzoites is indicated based on endogenous tagging by indirect immunofluorescence. Green indicates rabbit anti-HA antibody. Red indicates IMC1. Blue indicates DNA-specific dye with DAPI. Scale bar, 2 µm. (d) Western blot analysis of UBLCP1 in RHΔku80 and UBLCP1-6HA strains, and blots are probed with rabbit anti-HA antibody to visualize target bands. [file 13071_2025_6766_MOESM3_ESM.tif]

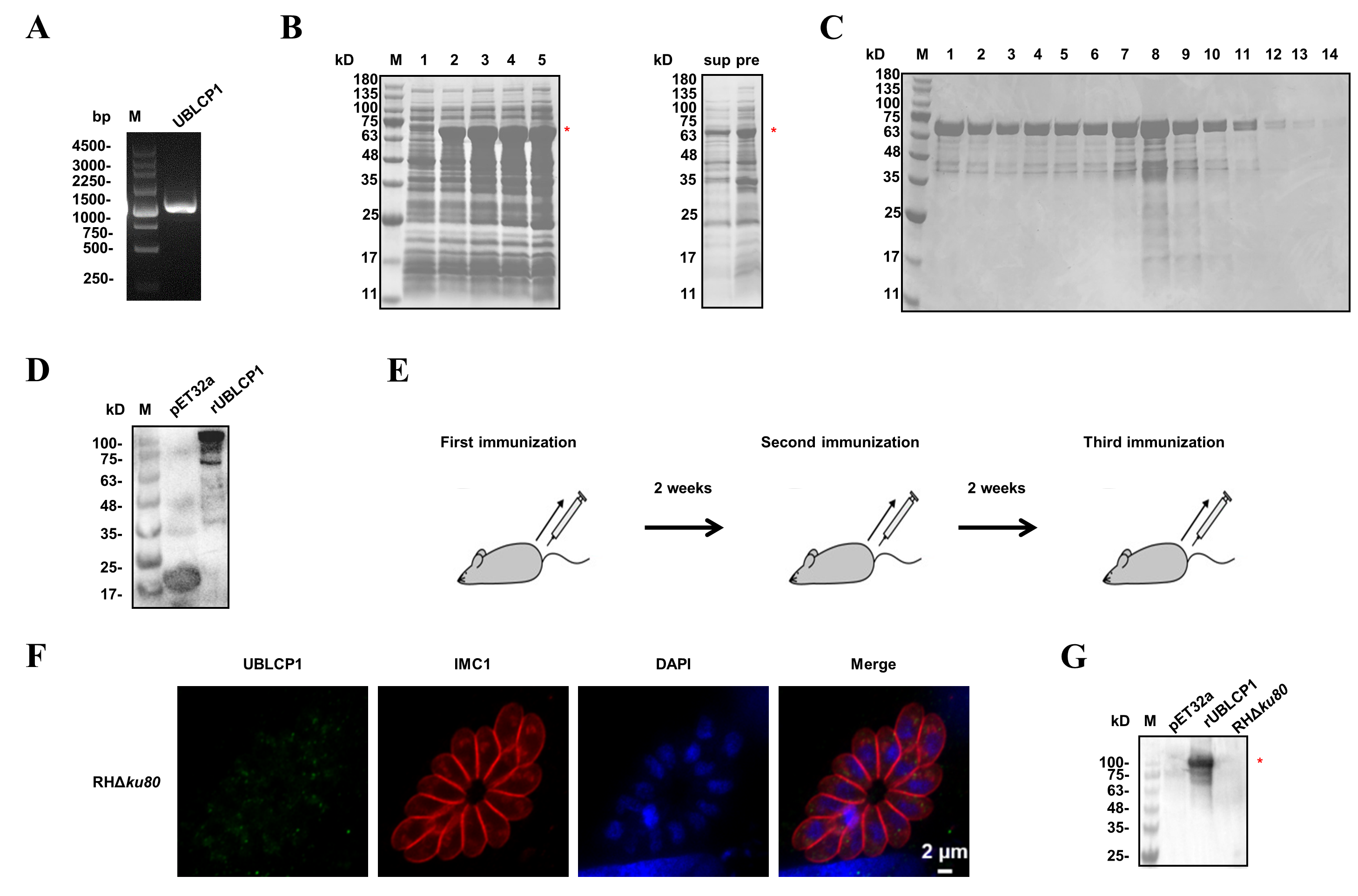

Supplement: Supplementary file 4 — Additional file 4: Figure S4. Localization of UBLCP1 in Toxoplasma gondii using mouse anti-UBLCP1 polyclonal antibodies. (a) PCR amplification of truncated fragment UBLCP1, which is ligated into the pET-32a(+) expression vector via the BamH I and Hind III restriction enzyme sites for protein expression in bacteria E. coli BL21 (DE3). (b) Prokaryotic expression of recombinant protein UBLCP1 (rUBLCP1) and SDS-PAGE. The optimal conditions for protein expression are 0.2 mM IPTG at 16°C for 12 h. M: Marker; Lanes 1, 2, 3, 4, 5 represent samples with different induction hours 0, 2, 4, 6, and 8 h, respectively; sup: supernatant; pre: precipitate. (c) Purification of recombinant UBLCP1. A gradient concentration of imidazole is used as an eluent. M: Marker; Lanes 1, 2, and 3 represent samples purified by 60 mM imidazole; Lanes 3-14 represent samples elucidated by 250 mM imidazole. (d) Western blot analysis of recombinant UBLCP1. Mouse anti-His antibody (1:1000) and goat anti-mouse IgG HRP-conjugated antibody (1:2000) are used as the primary and secondary antibodies, respectively. Lane 1: recombinant pET-32a protein as a control; Lane 2: recombinant UBLCP1. (e) Diagram illustrating the immunization process of mice using the purified recombinant UBLCP1. (f) The subcellular localization of UBLCP1 in T. gondii tachyzoites. Green indicates mouse anti-UBLCP1 polyclonal antibody. Red indicates IMC1. Blue indicates DNA-specific dye by DAPI. Scale bar, 2 µm. (g) Western blot analysis of recombinant pET32a protein, recombinant UBLCP1 and RHΔku80, blots are probed with mouse anti-UBLCP1 polyclonal antibodies. [file 13071_2025_6766_MOESM4_ESM.tif]

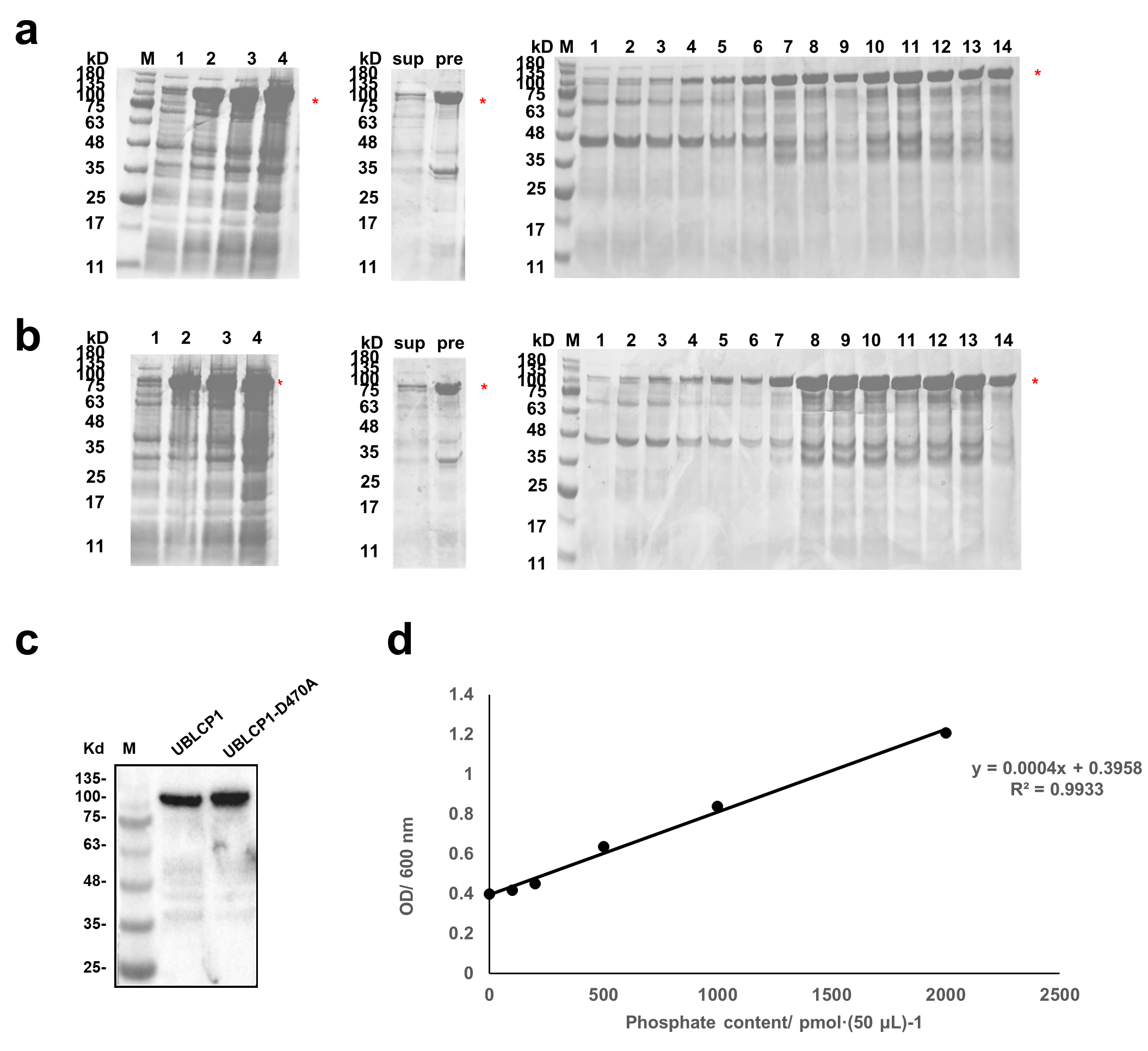

Supplement: Supplementary file 5 — Additional file 5: Figure S5. Determination of UBLCP1 phosphatase activity in vitro. (a) and (b) Induced expression, expression characteristic analysis and purification of recombinant protein UBLCP1 (a) or the mutation protein UBLCP1-D470A (b). The left panels show the induced expression of recombinant protein UBLCP1 or the mutation protein UBLCP1-D470A; M: Marker; 1, 2, 3, 4, 5 represent samples with different induction hours 0/2/4/6/8 h respectively. The middle panels show the obvious bands exclusively both in the insoluble fraction and the soluble fraction determined by SDS-PAGE results; sup: supernatant, pre: precipitate. The right panel shows the purification of UBLCP1 or UBLCP1-D470A protein using the elution buffer (25 mM tris-HCl, 300 mM NaCl, and 250 mM imidazole). M: Marker; 1–14 represent samples purified by the elution buffer. (c) Purified recombinant proteins identification by western blot using mouse anti-His antibody (1:1000) and goat anti-mouse IgG HRP-conjugated antibody (1:2,000). (d) Drawing of phosphate standard curve. The absorbance of standard solutions (phosphate content at 100, 200, 500, 1000 and 2000 pmol·(50 μL)-1) at 600 nm is determined spectrophotometrically using the molybdate dye method. In vitro phosphatase assay is carried out according to the manufacturer’s suggestion (Promega, USA). [file 13071_2025_6766_MOESM5_ESM.tif]
